# Newly Synthesized Dihydroxyphenyl–Nitroaryl Schiff Base Immobilized on Silica for Green UA-DMSPE of Toxic Metals in Marine Samples: Experimental Performance, DFT Insights, Molecular Docking, and In Silico Toxicological Evaluation

**DOI:** 10.1021/acsomega.6c00084

**Published:** 2026-04-22

**Authors:** Serkan Öncüoğlu, Taşkın Mumcu

**Affiliations:** † Department of Chemistry, Faculty of Sciences, 37508 Dokuz Eylul University, Merkez Campus, 35160 Buca-Izmir, Turkiye; ‡ Department of Chemistry, Faculty of Sciences, Dokuz Eylul University, Merkez Campus, 35160 Buca-Izmir, Turkiye

## Abstract

Heavy metal contamination of aquatic and marine environments continues to pose serious risks to ecosystems and human health, particularly through bioaccumulation in seafood matrices. In this study, a novel dihydroxyphenyl–nitroaryl azomethine (Schiff base) ligand (TSL1) was synthesized, structurally characterized, and immobilized onto a silica support to obtain a functionalized sorbent for ultrasound-assisted dispersive micro-solid-phase extraction (UA-DMSPE). The developed sorbent was applied for the selective extraction and preconcentration of Hg­(II), Cu­(II), Pb­(II), and Cd­(II) ions prior to flame atomic absorption spectrometric determination. Key experimental parameters affecting extraction efficiency were systematically optimized and statistically validated using one-way ANOVA. Under optimized conditions, the proposed UA-DMSPE method exhibited high recoveries (≥90%), low detection limits (0.60–1.50 μg L^–1^), good precision (RSD < 3.0%), and strong tolerance toward common matrix constituents. The method was successfully applied to seawater and mussel samples, demonstrating reliable performance with controlled matrix effects. Density functional theory (DFT) calculations provided insight into the electronic properties and coordination behavior of TSL1, indicating favorable and comparable affinity toward the investigated metal ions, in agreement with the experimental extraction results. Molecular docking studies provided complementary information on potential biological interaction profiles. In addition, in silico toxicity assessment suggested an acceptable preliminary safety profile for analytical applications, and green analytical metrics confirmed the environmental compatibility of the proposed approach. Overall, the developed TSL1-functionalized silica sorbent combined with UA-DMSPE offers an efficient, selective, and environmentally benign strategy for trace-level determination of toxic metals in complex marine and food-related matrices.

## Graphical abstract

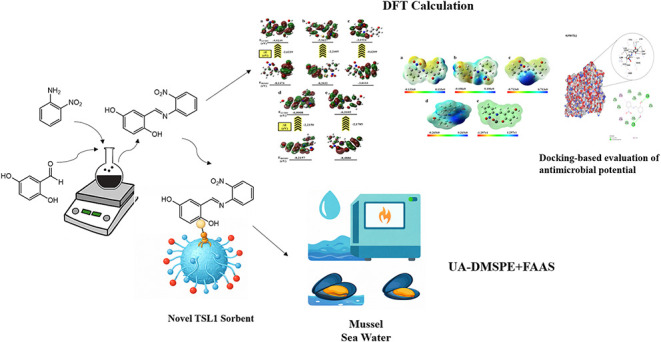

## Introduction

Heavy metal pollution has become one of the most significant environmental problems of modern times due to the nonbiodegradable nature and long-term persistence of these elements in ecosystems.^1^ In particular, mercury (Hg), lead (Pb), cadmium (Cd), and copper (Cu) exhibit high toxicity even at trace concentrations, accumulate in living tissues, and are transferred through the food chain, posing serious ecotoxicological risks.^2^ The release of these metals into marine environments through industrial effluents, agricultural runoff, domestic discharges, and atmospheric deposition leads to their accumulation in aquatic organisms and results in long-term threats to human health.^3^ Mussels are among the most reliable bioindicators of marine pollution because they are sessile filter feeders that readily accumulate metals present in their surrounding environment. However, the accumulation of toxic metals in mussel tissues can cause their transfer to humans through consumption, leading to irreversible damage to the nervous system, liver, kidneys, and reproductive organs.^4^ Therefore, the accurate, selective, and sensitive determination of toxic metals in mussel and seawater samples is of great importance for environmental monitoring and food safety.^5^ In real environmental samples, metal ions are often present at trace levels, making their direct determination difficult. For this reason, appropriate preconcentration and separation steps are generally required prior to instrumental analysis.^6^ Conventional techniques such as liquid–liquid extraction (LLE), cloud point extraction (CPE), coprecipitation, and solid-phase extraction (SPE) have been widely employed; however, these approaches often involve long extraction times, high solvent consumption, and multiple operational steps.^7^ To address these limitations, dispersive micro-solid-phase extraction (DMSPE) has emerged as an attractive alternative owing to its low sorbent requirement, short extraction time, and high enrichment efficiency. Furthermore, ultrasound-assisted dispersive micro-solid-phase extraction (UA-DMSPE) enhances mass transfer between the sorbent and analyte, leading to improved extraction efficiency and reproducibility.^8^ Schiff base ligands have attracted considerable attention in recent years in the development of functionalized sorbent materials due to their strong chelating ability toward metal ions through azomethine nitrogen and donor oxygen atoms. Numerous studies have reported the successful immobilization of Schiff base derivatives onto various solid supports, including silica, polymeric matrices, and magnetic nanoparticles, for the selective extraction and preconcentration of toxic metal ions from environmental and food samples. These materials have demonstrated high selectivity, chemical stability, and satisfactory reusability, highlighting the effectiveness of Schiff base-functionalized sorbents in microextraction and solid-phase extraction applications. The presence of tunable functional groups in Schiff base structures allows the modulation of coordination strength and selectivity toward specific target metals, making them promising candidates for environmentally friendly analytical systems.^9^ In this study, a novel Schiff base ligand derived from (*E*)-2-(((2-nitrophenyl)­imino)­methyl)­benzene-1,4-diol was synthesized for the first time and covalently immobilized onto a silica surface to obtain a functionalized sorbent, hereafter referred to as TSL1. The developed material was applied to the UA-DMSPE of Hg­(II), Cu­(II), Pb­(II), and Cd­(II) ions in mussel and seawater samples collected from the İnciraltı region (İzmir, Türkiye) prior to FAAS determination. To support the experimental findings and to gain insight into the metal–ligand interaction mechanism, complementary theoretical investigations were performed. To provide molecular-level insight into the coordination behavior of the synthesized ligand and to preliminarily assess its safety profile, complementary computational investigations were performed. Density functional theory (DFT) calculations were employed to examine the electronic characteristics relevant to metal binding, while molecular docking analyses and in silico toxicity prediction were conducted to support mechanistic interpretation and preliminary safety considerations. These approaches were used as supportive tools to reinforce the experimental findings rather than as primary outcomes of the study. The environmental performance of the proposed method was evaluated using green analytical metrics, including the Analytical Eco-Scale, GAPI, and AGREE, confirming the eco-friendly character of the developed procedure. Matrix effect studies demonstrated the robustness and selectivity of the method in complex marine matrices, while optimization parameters were statistically validated using one-way ANOVA, ensuring reliable and reproducible results. Overall, the developed UA-DMSPE method provides a sensitive, selective, and environmentally compatible approach for the determination of trace-level heavy metals in marine environments. To the best of our knowledge, this study represents the first application of this Schiff base ligand as a functionalized silica sorbent for the UA-DMSPE of Hg­(II), Cu­(II), Pb­(II), and Cd­(II) ions from real mussel and seawater samples, supported by combined experimental, computational, and toxicological evaluations. The novelty of this study lies in the first-time synthesis and immobilization of a dihydroxyphenyl–nitroaryl azomethine Schiff base onto a silica support for use as a functionalized sorbent in UA-DMSPE. Unlike previously reported Schiff base–based sorbents, the present material integrates a dihydroxyphenyl moiety and a nitroaryl group, providing multiple donor sites that enhance coordination toward Hg­(II), Cu­(II), Pb­(II), and Cd­(II) ions. The designed system addresses key analytical challenges associated with trace-level metal determination in complex marine and biological matrices by offering rapid extraction, high selectivity, low detection limits, and strong matrix tolerance under environmentally benign conditions. In addition, the combined use of experimental evaluation with DFT calculations, molecular docking, and in silico toxicity assessment provides a comprehensive understanding of both analytical performance and molecular interaction behavior, which has rarely been reported for UA-DMSPE-based systems.

## Experimental Section

### Chemicals and Reagents

All reagents and solvents used throughout the study were of analytical grade and were obtained from Merck, Sigma-Aldrich, Fluka, or Riedel-de Haën. Silica gel (70–230 mesh) was used as the solid support material for sorbent preparation. Metal salts employed for analytical studies, including HgCl_2_, PbCl_2_, CdCl_2_, and CuCl_2_, were purchased from Merck or Sigma-Aldrich. Stock solutions of Hg­(II), Pb­(II), Cd­(II), and Cu­(II) (1000 mg L^–1^) were prepared in ultrapure water and stored at +4 °C. Hg­(II) stock solutions were freshly prepared and stored under acidic conditions to ensure stability. Working standard solutions were freshly prepared on a daily basis by appropriate dilution of the stock solutions with ultrapure water. Nitric acid (HNO_3_) and hydrochloric acid (HCl), used for pH adjustment and elution studies, were of analytical grade. Ultrapure water (resistivity ≥ 18.2 MΩ cm) used in all experiments was supplied by a Thermo Scientific Smart2Pure Pro ultrapure water purification system. The melting points of the synthesized compounds were measured using a digital electrothermal melting point apparatus (Gallenkamp), with samples placed in sealed capillary tubes. The synthesized compound was structurally characterized using spectroscopic and analytical techniques. ^1^H NMR spectra were acquired on a Bruker WH-400 Fourier transform NMR spectrometer. Infrared measurements were performed using a PerkinElmer Spectrum BX-II FT-IR spectrometer. Elemental analysis for carbon, hydrogen, and nitrogen was carried out with a LECO CHNS-O-9320 elemental analyzer. The surface morphology and elemental composition of the synthesized sorbent were examined by scanning electron microscopy coupled with energy-dispersive X-ray spectroscopy (SEM/EDX) using a Zeiss Evo HD15 instrument equipped with a tungsten electron source. Textural properties of the bare and functionalized silica sorbents, including specific surface area, pore volume, and pore size distribution, were determined by Brunauer–Emmett–Teller (BET) nitrogen adsorption–desorption analysis using a Micromeritics Gemini VII Series surface area analyzer. Ultrasound-assisted dispersive micro-solid-phase extraction (UA-DmSPE) procedures were carried out in a Bandelin ultrasonic bath. Quantitative determination of Hg­(II), Pb­(II), Cd­(II), and Cu­(II) ions was performed using a PerkinElmer AAnalyst 700 flame atomic absorption spectrometer (FAAS) equipped with hollow cathode lamps. The analytical wavelengths used for metal determination were 253.7 nm for Hg­(II), 217.0 nm for Pb­(II), 228.8 nm for Cd­(II), and 324.8 nm for Cu­(II), selected according to literature data and manufacturer recommendations. Instrumental parameters were optimized individually for each metal ion to ensure maximum sensitivity and signal stability.

### Real Samples Preparation

Seawater samples were collected in precleaned polyethylene bottles, immediately acidified to pH ≈ 2 with ultrapure HNO_3_ to prevent metal adsorption and microbial activity, and stored at 4 °C until analysis. Prior to extraction, seawater samples were allowed to reach room temperature and adjusted to the optimum pH of the developed UA-DMSPE method using dilute HNO_3_ or NaOH solutions; the pH was kept below 6.0 to avoid hydrolysis and possible loss of Hg­(II).^10^ Mussel samples were transported to the laboratory under cold conditions, thoroughly rinsed with ultrapure water to remove surface contaminants, and the soft tissues were carefully separated using plastic tools. The edible tissues were homogenized, and an accurately weighed portion (0.20–0.50 g) was subjected to wet acid digestion using concentrated HNO_3_, with the optional addition of H_2_O_2_ to ensure complete oxidation of the organic matrix. Digestion was carried out under controlled heating until a clear solution was obtained, after which the digest was cooled, quantitatively transferred, and diluted to a known volume with ultrapure water.^11^ An aliquot of the digested mussel solution was then adjusted to the optimum extraction pH (maintained below pH 6.0 for Hg­(II)) and subjected to the UA-DMSPE procedure under the same conditions as those optimized for aqueous standards. All real samples were analyzed in triplicate together with procedural blanks and spiked samples to evaluate recovery and matrix effects.

### Synthesis of (*E*)-2-(((2-Nitrophenyl)­imino)­methyl)­benzene-1,4-diol (TSL1)

The dihydroxyphenyl–nitroaryl azomethine (Schiff base) ligand was synthesized following a literature-reported condensation protocol.^12^ In a typical synthesis, 2,5-dihydroxybenzaldehyde (1.0 equiv) was dissolved in absolute ethanol (50 mL) under heating, and the reaction medium was acidified with a few drops of concentrated sulfuric acid. An equimolar amount of 2-nitroaniline (1.0 equiv) was then added to the solution. The resulting mixture was refluxed for 4 h under continuous stirring. After completion of the reaction, the mixture was allowed to cool to room temperature, leading to the formation of a solid precipitate. The product was collected by filtration, washed several times with ethanol, and dried under vacuum to yield the target Schiff base ligand as a solid. Yield: 78%; yellow powder; mp 189 °C. (Figure 1).

**Figure 1:**
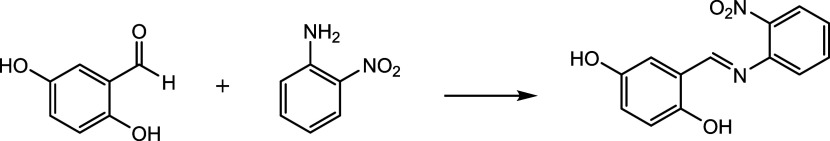
Synthesis of (*E*)-2-(((2-nitrophenyl)­imino)­methyl)­benzene-1,4-diol (TSL1).

### Preparation of the Sorbent

Prior to functionalization, silica gel was activated by washing with 0.5 mol L^–1^ HNO_3_ to remove surface impurities and trace metal contaminants, followed by extensive rinsing with ultrapure water until neutral pH was reached. The activated silica was then dried and used for ligand immobilization. For sorbent preparation, 1 g of the pretreated silica was mixed with 10 mL of a 1 × 10^–4^ mol L^–1^ solution of the synthesized Schiff base ligand in chloroform, and the suspension was stirred at room temperature for 24 h to promote effective interaction between the ligand molecules and the surface silanol groups of silica. After immobilization, the solid material was separated by filtration using a sintered glass funnel, thoroughly washed with chloroform and ultrapure water to remove any physically adsorbed or unbound ligand, and dried at room temperature.^13^ The resulting ligand-functionalized silica sorbent was stored in a desiccator and subsequently characterized by FT-IR, SEM, BET surface area analysis, solid-state ^13^C NMR, ^1^H NMR of the ligand, and elemental analysis to confirm successful immobilization and surface modification before its application in UA-DMSPE experiments.

### Ultrasound-Assisted Dispersive Micro-Solid-Phase Extraction Procedure (UA-DMSPE)

Optimization studies were initially performed using standard aqueous solutions of the target metal ions. Following the determination of the optimum extraction conditions, the developed UA-DMSPE procedure was subsequently applied to real samples under the same optimized parameters. For extraction, 10.0 mL of the solution was transferred into a polypropylene centrifuge tube, and 40 mg of the ligand-functionalized silica sorbent was added. The pH of the medium was adjusted to the optimum value using dilute HNO_3_ or NaOH solutions; in samples containing Hg­(II), the pH was carefully maintained below 6.0 to avoid hydrolysis and possible loss of mercury species. The resulting suspension was subjected to ultrasonication in an ultrasonic bath at room temperature for the optimized extraction time to ensure efficient dispersion of the sorbent and rapid adsorption of the metal ions. After extraction, phase separation was achieved by centrifugation at 5000 rpm for 10 min, and the supernatant was carefully decanted. Elution of the retained metal ions was carried out by adding 1.0 mL of 1.0 mol L^–1^ HNO_3_ onto the collected sorbent, followed by a second ultrasonication step. The mixture was subsequently centrifuged again under the same conditions (5000 rpm for 10 min), and the obtained eluate was collected for FAAS determination.^14^ The same procedure was applied to pretreated seawater and digested mussel samples without modification of the optimized conditions. All experiments were conducted in triplicate together with procedural blanks and spiked samples. It should be noted that the present method is based on ultrasound-assisted dispersive micro-solid-phase extraction performed in batch mode (UA-DMSPE), and no packed-column procedure was applied. Therefore, adsorption and equilibration processes were evaluated through the optimized UA-DMSPE parameters, where the ultrasonication extraction time represents the contact/equilibration time between the sorbent and metal ions. Desorption of the retained metals was achieved using nitric acid under ultrasonication, followed by centrifugation and FAAS determination. The selectivity of the method was assessed by interference and matrix tolerance studies, while regeneration and reusability were evaluated by consecutive adsorption–desorption cycles.

### Protein Modeling

The selected protein targets (1BTL, 1KE4, 1MWS, and 3MZD) belong to clinically relevant β-lactamase families associated with bacterial antibiotic resistance, whereas 4UYM and 5TZ1 correspond to lanosterol 14α-demethylase (CYP51), a key enzyme involved in fungal ergosterol biosynthesis and antifungal resistance mechanisms. These proteins were selected based on their biological significance, availability of high-resolution crystal structures, and their frequent use in structure-based molecular docking investigations.^15,16^

Three-dimensional structural models of the proteins corresponding to PDB IDs 1BTL, 1KE4, 1MWS, 3MZD, 4UYM, and 5TZ1 were generated using the BIOVIA Discovery Studio platform. For each protein target, crystallographic templates offering the highest sequence coverage together with the most favorable (lowest) resolution were carefully selected in order to maximize structural accuracy and reliability. As illustrated in Figure 2, the constructed models display notable differences in overall conformation and molecular architecture, which can be attributed to variations in amino acid composition and intramolecular interaction networks.

**Figure 2:**
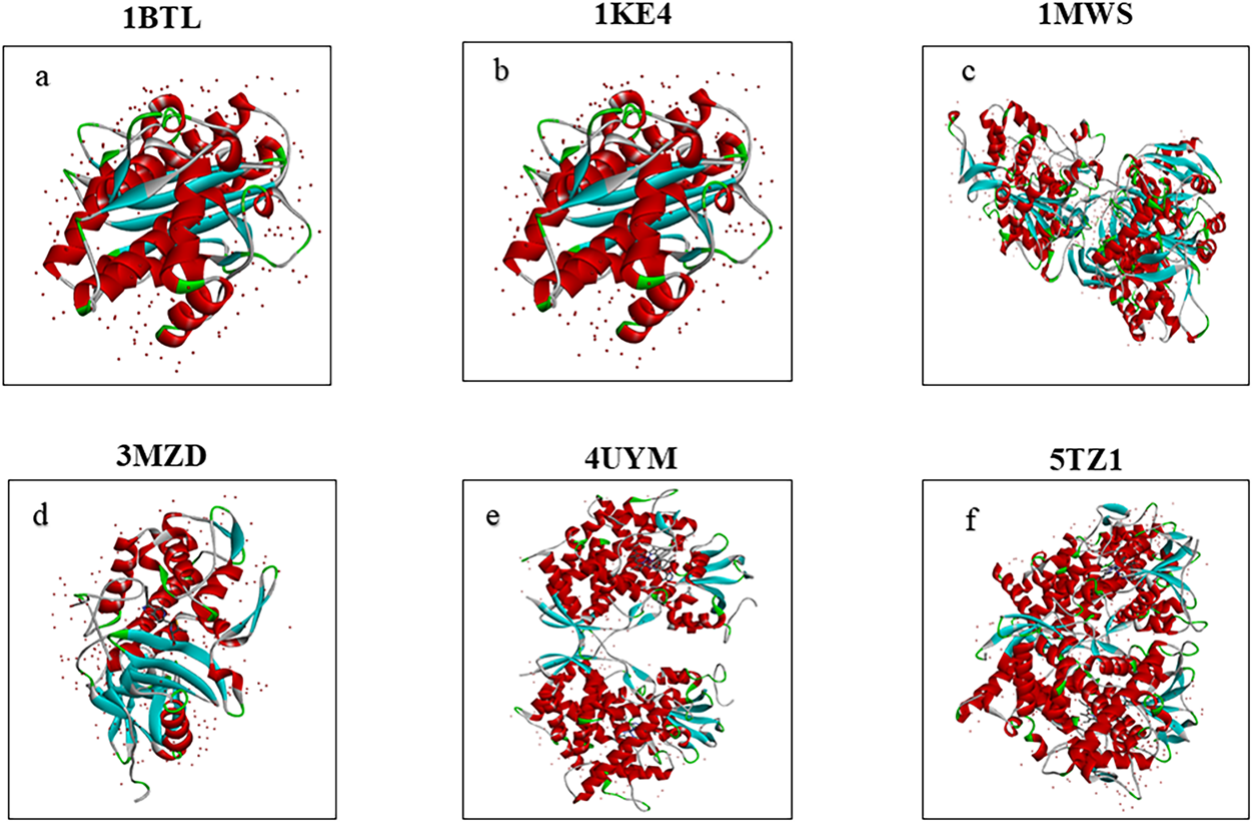
Modeled 3D structures of target proteins: (a) 1BTL, (b) 1KE4, (c) 1MWS, (d) 3MZD, (e) 4UYM, and (f) 5TZ1, generated using BIOVIA Discovery Studio Visualizer 2024.

### Molecular Docking

Following the completion of three-dimensional protein modeling, molecular docking calculations were performed for all six targets using the CB-Dock2 web server.^17^ In these simulations, (*E*)-2-(((2-nitrophenyl)­imino)­methyl)­benzene-1,4-diol (TSL1) was employed as the ligand molecule. CB-Dock2 applies a cavity-based recognition strategy to automatically locate accessible binding pockets on the protein surface and subsequently estimates the binding affinity of the ligands within these regions. For each protein–ligand system, the server ranks the predicted binding sites and reports the five cavities associated with the most favorable (lowest) binding energy values, which are considered the most likely interaction regions.

### Density Functional Theory (DFT)

Density Functional Theory (DFT) calculations were performed to investigate the electronic structure and fundamental physicochemical properties of the synthesized TSL1 ligand and its corresponding metal complexes.^18^ Molecular structures were initially constructed and preoptimized using ChemDraw and Avogadro, while visualization and orbital analyses were carried out with GaussView 5.0. All quantum-chemical calculations and full geometry optimizations were conducted using the Gaussian 09W program package.^19^ The B3LYP hybrid functional was employed throughout the study. The 6–311G basis set was applied for nonmetal atoms (C, N, O, and H), whereas the LANL2DZ basis set with associated effective core potentials (ECPs) was used for the metal centers (Hg, Pb, Cd, and Cu). The use of ECPs enables an efficient treatment of core electrons and provides an approximate inclusion of scalar relativistic effects, which is particularly important for heavy metal atoms. This mixed basis set approach offers a balanced compromise between computational cost and reliable description of metal–ligand interactions.^20^ Molecular electrostatic potential (MEP) maps were generated to examine the charge distribution over the molecular surface and to identify regions susceptible to electrophilic and nucleophilic interactions. In these maps, areas exhibiting positive electrostatic potential, which are more favorable for nucleophilic attack, are depicted in blue, whereas regions with pronounced negative potential, preferred for electrophilic interactions, appear in red. Zones with intermediate potential values are shown in green, offering a clear visualization of weakly polarized regions and potential reactive sites within the molecular framework.^21^ To gain further insight into the electronic behavior of the ligand system, Frontier Molecular Orbital (FMO) analysis was carried out with particular emphasis on the energies and spatial distributions of the HOMO and LUMO orbitals.^22^ The HOMO energy level is associated with the electron-donating capability of the ligand, while the LUMO reflects its electron-accepting character. Together, these orbitals provide essential information regarding molecular reactivity, kinetic stability, and possible charge-transfer processes, especially in relation to coordination tendencies and ligand–metal interactions.^23^ The HOMO–LUMO energy gap (Δ*E*), defined as the energy difference between the HOMO and LUMO levels, was evaluated as a key descriptor of electronic stability and chemical reactivity. A larger Δ*E* value is generally indicative of enhanced thermodynamic stability and lower chemical reactivity, whereas a smaller gap suggests increased polarizability and a higher propensity for electronic transitions. In addition, global reactivity descriptors, including ionization potential, electron affinity, electronegativity, chemical hardness, softness, chemical potential, and electrophilicity index, were calculated using well-established theoretical relationships reported in the literature,^24^ providing a comprehensive quantum-chemical interpretation of the electronic properties of the investigated

1 $${\Delta E}_{\mathrm{gap}}=\left|E_{\mathrm{HOMO}}-E_{\mathrm{LUMO}}\right|$$

2 $$I=-E_{HOMO}$$

3 $$\mathrm{EA}=-E_{\mathrm{LUMO}}$$

4 $$\chi =\frac{I+\mathrm{EA}}{2}$$

5 $$\eta =\frac{E_{\mathrm{LUMO}}-E_{\mathrm{HOMO}}}{2}$$

6 $$\sigma =\frac{1}{2\eta }$$

7 $$\mu =\frac{E_{\mathrm{HOMO}}+E_{\mathrm{LUMO}}}{2}$$

8 $$\omega =\frac{\mu^{2}}{2\eta }$$

### Toxicity

A theoretical toxicity analysis of synthesized TSL1 was conducted using the Protox 3.0 web server.

## Results and Discussion

### Characterization of (*E*)-2-(((2-Nitrophenyl)­imino)­methyl)­benzene-1,4-diol (TSL1)

To elucidate the chemical structure, surface morphology, and textural properties of the synthesized sorbent, comprehensive characterization studies were performed using FT-IR spectroscopy, SEM–EDX analysis, BET surface area measurements, ^1^H NMR spectroscopy, and elemental analysis. These complementary techniques collectively provided detailed information on the successful functionalization of the silica surface, the preservation of the ligand structure, and the morphological and compositional features of the resulting sorbent.

### FT-IR Characterization

The successful functionalization of the silica surface with the TSL1 ligand was confirmed by FT-IR spectroscopy (Figure 3). The FT-IR spectrum of bare silica (black) exhibits a broad absorption band at approximately 3448 cm^–1^, attributed to the stretching vibrations of surface silanol (O–H) groups. The intense band observed near 1100 cm^–1^ corresponds to the asymmetric stretching vibrations of Si–O–Si linkages, which constitute the backbone of the silica framework. Additional characteristic silica bands were detected at around 970 cm^–1^, associated with Si–OH/Si–O vibrations, 794 cm^–1^ corresponding to symmetric Si–O–Si vibrations, and 467 cm^–1^, assigned to Si–O bending modes, confirming the structural integrity of the silica support. The FT-IR spectrum of the TSL1 ligand (blue) displays a broad band centered at approximately 3360 cm^–1^, which can be attributed to the stretching vibrations of phenolic O–H groups, possibly enhanced by intermolecular hydrogen bonding. A prominent absorption band at 1623 cm^–1^ is assigned to the C=N (imine) stretching vibration, representing the characteristic structural feature of the Schiff base ligand. Aromatic ring vibrations appear clearly in the 1596–1444 cm^–1^ region, while the presence of the nitro-substituted aromatic moiety is supported by characteristic −NO_2_ vibrations, particularly the asymmetric stretching near 1505 cm^–1^ and the symmetric stretching bands in the 1348–1384 cm^–1^ range. Moreover, absorption bands observed between 1254 and 1212 cm^–1^ are attributed to C–O and/or C–N stretching vibrations, whereas the bands at 855–851, 777, 745, and ∼699 cm^–1^ correspond to out-of-plane bending vibrations of aromatic C–H groups. In the FT-IR spectrum of the functionalized silica material (green), characteristic absorption bands originating from both the silica matrix and the TSL1 ligand are simultaneously observed, confirming successful surface modification. The persistence of the diagnostic C=N stretching band at approximately 1625 cm^–1^ indicates that the ligand retains its structural integrity after immobilization onto the silica surface. The broad band observed around 3285 cm^–1^ can be attributed to overlapping O–H stretching vibrations from surface silanol groups and phenolic hydroxyl groups of the ligand, suggesting enhanced hydrogen-bonding interactions upon functionalization. Furthermore, the appearance of ligand-related bands in the 1200–1300 cm^–1^ region, together with the preserved Si–O–Si vibrations of the silica framework, provides strong evidence for the successful immobilization of the TSL1 ligand onto the silica surface without disruption of the silica structure. Overall, the coexistence of silica-specific bands and ligand-derived diagnostic absorptions in the FT-IR spectrum of the silica–TSL1 sorbent unequivocally confirms the successful functionalization of the silica surface with the TSL1 ligand.

**Figure 3:**
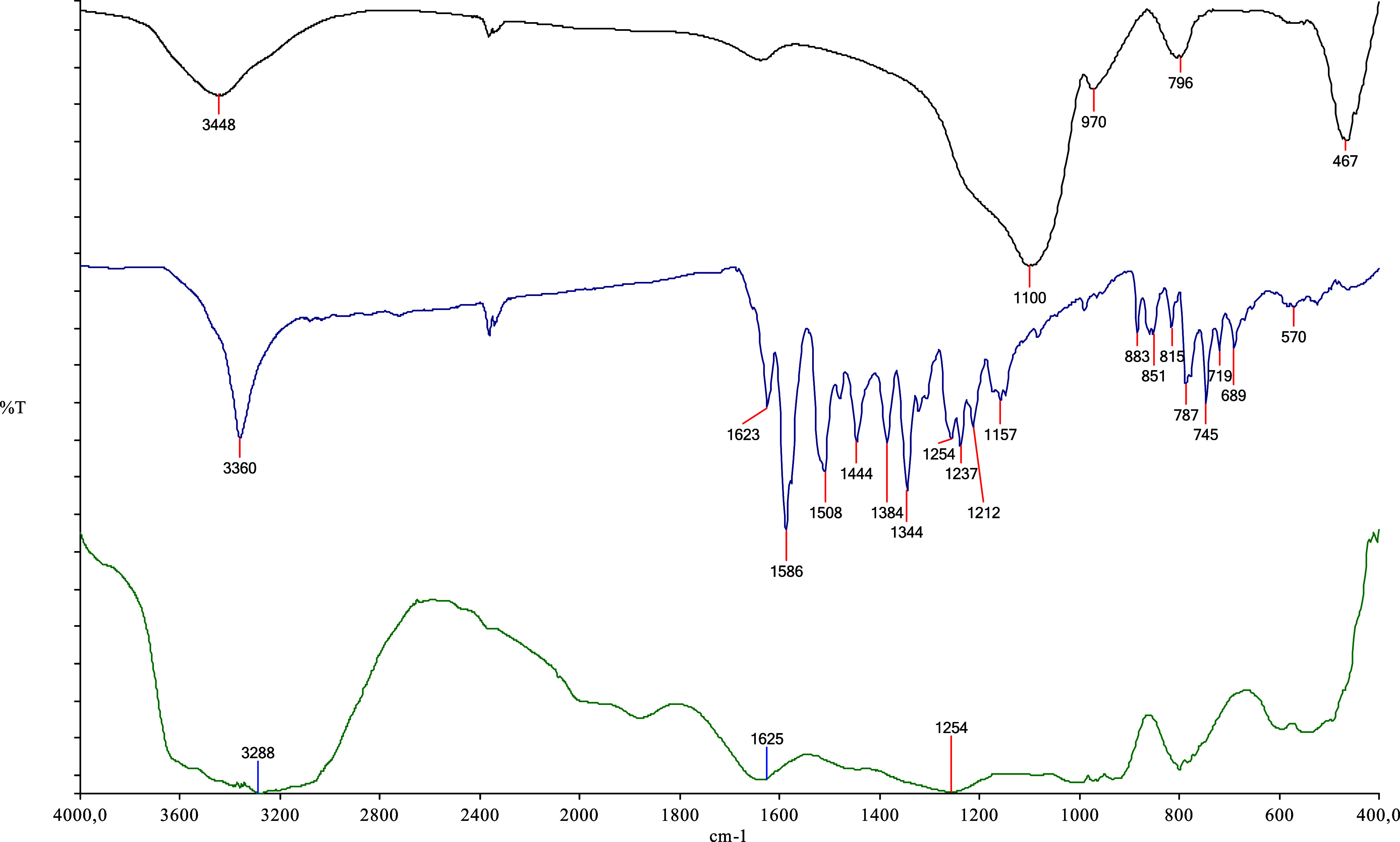
FT-IR spectra of bare silica (black), TSL1 ligand (blue), and TSL1-functionalized silica sorbent (green).

The FT-IR spectrum (KBr pellet) of the synthesized Schiff base (TSL1) exhibited characteristic absorption bands at ν /cm^–1^ 3360 (O–H), 1623 (C=N), 1508 and 1384 (NO_2_), 1254 (C–O), and 1212 (C–N).

### NMR Characterization and Elemental Analysis

C_13_H_10_N_2_O_4_: Calc. %C: 60.48; H, 3.89; N, 10.85 Found: %C: 60.32: H, 3.81; N, 10.87. ^1^H NMR (400 MHz, DMSO-*d*_6_) δ 11.77 (s, 1H), 9.38 (s, 1H), 8.84 (s, 1H), 8.00 (d, *J* = 7.5, 1H), 7.72 (dd, *J* = 7.5, 1.5 Hz, 1H), 7.53 (dd, *J* = 7.5, 1.5 Hz, 1H), 7.42 (d, *J* = 7.5, 1H), 7.01 (s, 1H), 6.79 (d, *J* = 7.5 Hz, 1H), 6.74 (d, *J* = 7.5, 1H) (Figure 4).

**Figure 4:**
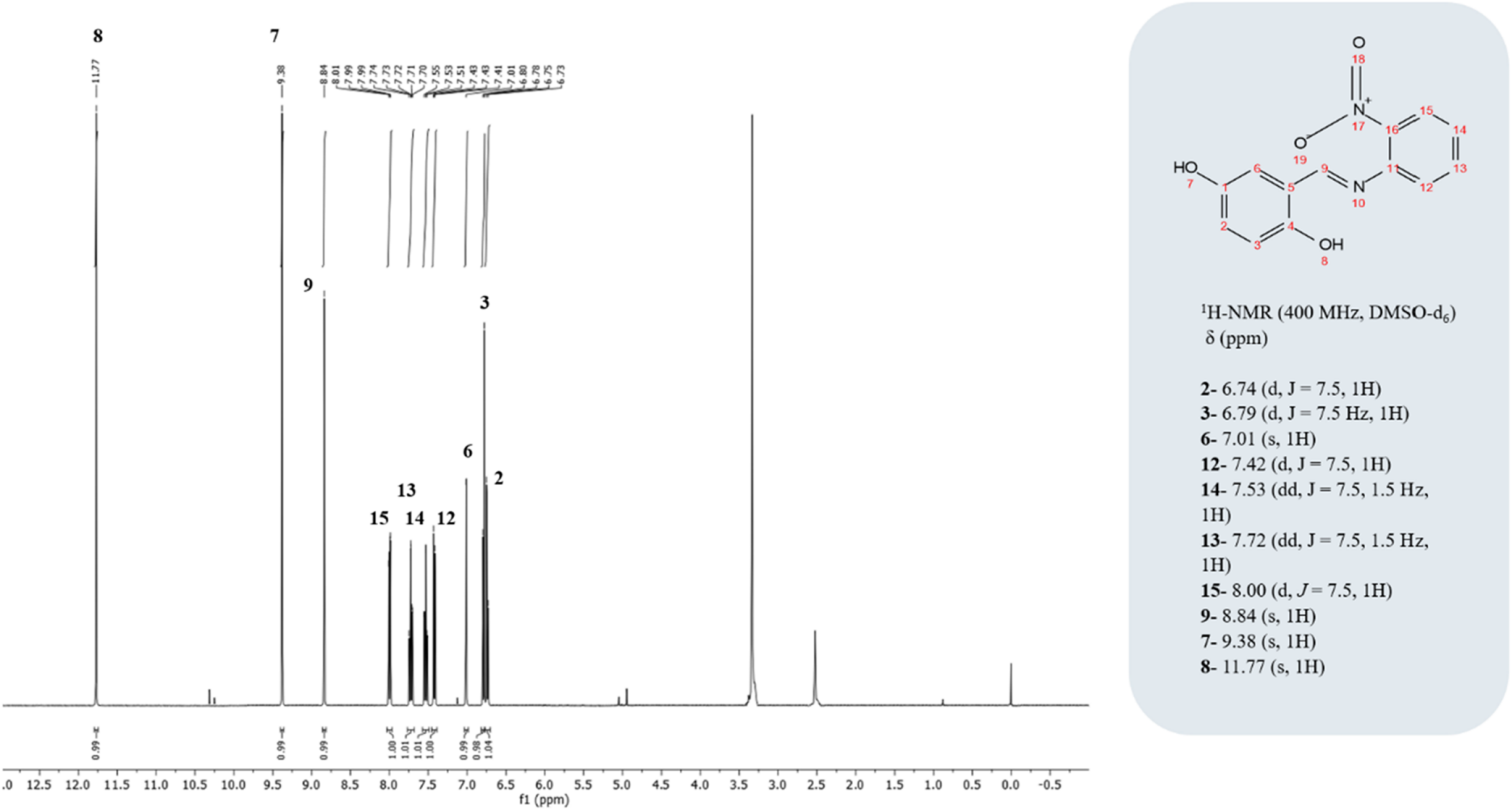
^1^H NMR spectrum of the synthesized TSL1 ligand.

### Surface Morphology of the Sorbent

The surface morphology of the sorbent before and after metal loading was investigated by SEM analysis, and the distribution of metal ions on the sorbent surface was evaluated by SEM–EDX spectra. As shown in Figure 5A, the metal-unloaded sorbent exhibits an irregular, layered, and rough surface morphology with fractured plate-like structures, providing a large surface area and numerous potential active sites for metal ion interaction. The corresponding EDX spectrum of the unloaded sorbent confirms the presence of the main matrix elements (Si and O), with no detectable signals of the target metal ions, indicating the absence of metal contamination prior to the adsorption process. After the metal loading procedure, the SEM image of the sorbent (Figure 5B) reveals that the overall surface morphology is largely preserved, while slight surface coverage and textural changes can be observed, suggesting the successful interaction of metal ions with the sorbent surface.^25^ The EDX spectrum of the metal-loaded sorbent clearly shows the appearance of characteristic signals corresponding to Pb, Cd, Cu, and Hg ions, which are absent in the unloaded sample. The emergence of these metal peaks provides strong evidence for the effective immobilization of the target metal ions onto the sorbent surface. The preservation of the sorbent morphology after metal loading further indicates that the adsorption process occurs without structural degradation, supporting the stability and reusability of the sorbent.

**Figure 5:**
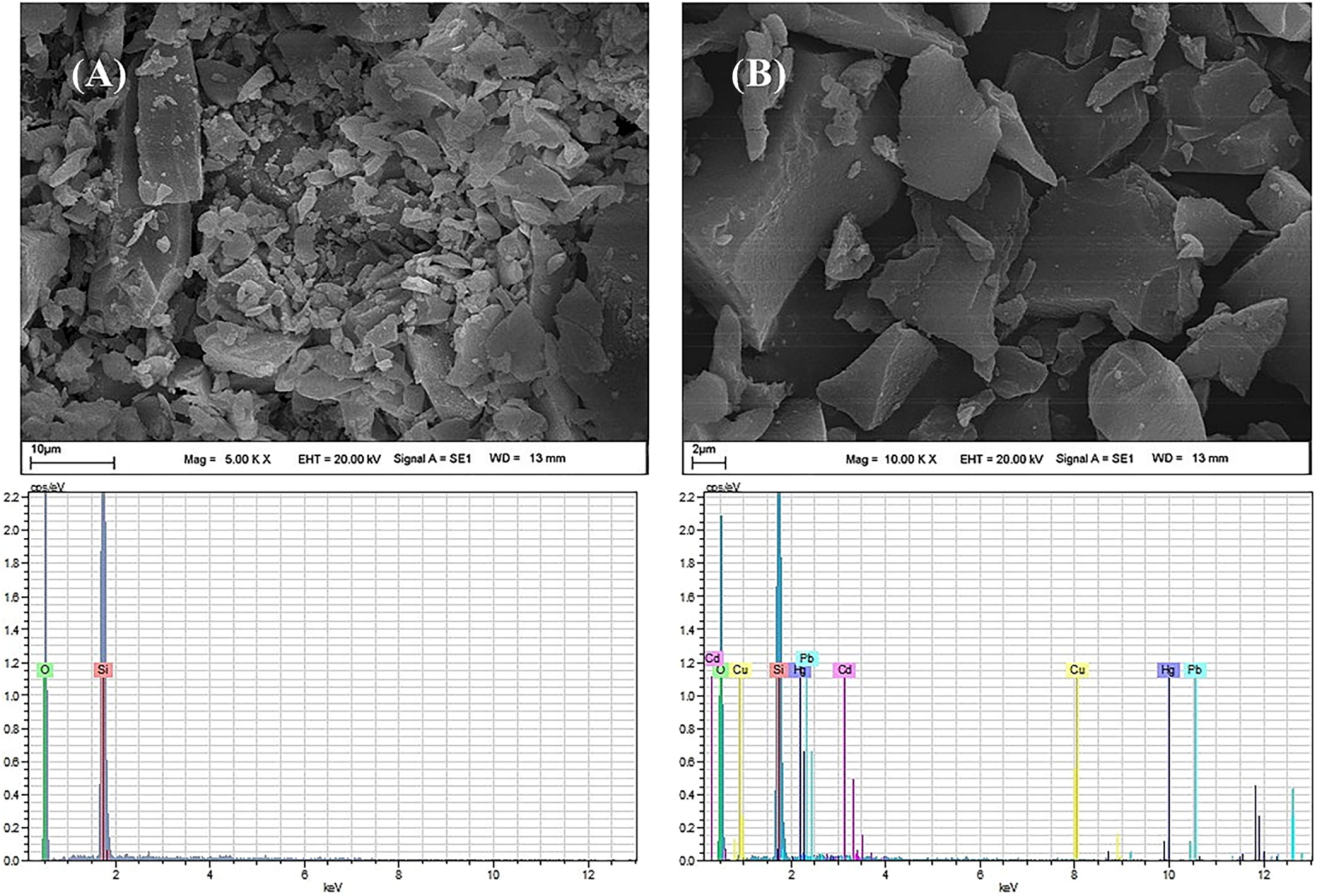
SEM images (A, B) and corresponding EDX spectra of the sorbent before and after metal loading: (A) SEM image and EDX spectrum of the sorbent before metal loading and (B) SEM image and EDX spectrum of the sorbent after metal loading.

### BET Surface Area Analysis

The surface textural properties of the metal-unloaded sorbent and metal-loaded sorbent were comparatively evaluated by nitrogen adsorption–desorption measurements using the Brunauer–Emmett–Teller (BET) method. As illustrated in Figure 6A, the metal-unloaded sorbent exhibits a typical type IV adsorption–desorption isotherm with a pronounced hysteresis loop, characteristic of a mesoporous structure with high surface accessibility. The corresponding BET results reveal a high specific surface area (*S*_BET_ = 469.24 m^2^ g^–1^) and a large total pore volume (0.72485 cm^3^ g^–1^), confirming the highly porous nature of the pristine sorbent support. After metal loading, the nitrogen adsorption–desorption isotherm of the metal-loaded sorbent (Figure 6B) retains the overall mesoporous profile; however, a marked reduction in the amount of adsorbed nitrogen is observed over the entire relative pressure range. As summarized in Table 1, the BET surface area decreases significantly from 469.24 to 272.76 m^2^ g^–1^, while the total pore volume declines from 0.72485 to 0.45830 cm^3^ g^–1^ after metal loading. These reductions, corresponding to a decrease of more than 40% in surface area and approximately 35% in pore volume, provide strong evidence for the successful immobilization of metal ions onto the silica surface. Consistent decreases are also observed in the Langmuir surface area, t-plot external surface area, and BJH adsorption–desorption surface areas, further confirming partial surface coverage and pore blockage induced by the incorporated metal species. The interaction of Hg­(II), Pb­(II), Cu­(II), and Cd­(II) ions with surface silanol (Si–OH) groups is expected to occur both on the external surface and within the mesoporous framework, leading to restricted nitrogen diffusion during adsorption–desorption measurements. Moreover, the slight increase in average pore diameter for the metal-loaded sorbent suggests preferential occupation or blocking of smaller pores by heavy metal ions, resulting in an apparent shift of the pore size distribution toward larger pore widths. Overall, the pronounced reductions in surface area and pore volume, together with the preservation of the mesoporous structure, clearly demonstrate the effective immobilization of Hg­(II), Pb­(II), Cu­(II), and Cd­(II) ions on the sorbent support. These results confirm that the developed silica-based sorbent possesses suitable surface and textural characteristics for the efficient retention of heavy metal ions.

**Figure 6:**
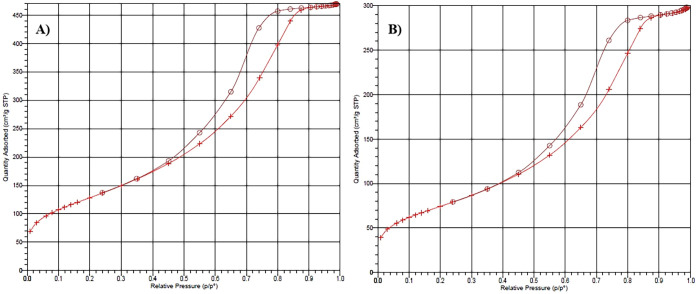
N_2_ adsorption–desorption isotherms of the sorbent before and after metal loading: (A) metal-unloaded sorbent, (B) metal-loaded sorbent.

**Table 1:** Surface Area and Pore Structure Parameters of the Sorbent before and after Metal Loading

parameter	sorbent (unloaded)	sorbent–metal-loaded
BET surface area (m^2^ g^–1^)	469.24	272.76
Langmuir surface area (m^2^ g^–1^)	690.68	400.89
t-Plot external surface area (m^2^ g^–1^)	477.56	280.83
BJH adsorption surface area (m^2^ g^–1^)	528.98	322.22
BJH desorption surface area (m^2^ g^–1^)	601.03	364.66
Total pore volume (cm^3^ g^–1^)	0.72485	0.45830
BJH adsorption pore volume (cm^3^ g^–1^)	0.75722	0.47671
BJH desorption pore volume (cm^3^ g^–1^)	0.75305	0.47537
Average pore width (BET, Å)	61.79	67.21
Average pore width (BJH adsorption, Å)	57.26	59.18
Average pore width (BJH desorption, Å)	50.12	52.14

### Proposed Adsorption/Extraction Mechanism

The adsorption mechanism of Hg­(II), Cu­(II), Pb­(II), and Cd­(II) onto the TSL1-functionalized silica sorbent is mainly governed by coordination interactions between the metal ions and the donor atoms of the immobilized Schiff base ligand. The azomethine nitrogen (−C=N−) and phenolic oxygen atoms provide electron-rich sites capable of forming stable chelate complexes with the target metal ions. At the optimum pH (6.0), partial deprotonation of the phenolic −OH groups enhances the availability of coordination sites, while the metal ions remain predominantly in their soluble ionic forms, favoring efficient complexation. Ultrasound irradiation promotes rapid dispersion of the sorbent and accelerates mass transfer, enabling fast attainment of adsorption equilibrium. The DFT results, including HOMO–LUMO and MEP analyses, support the strong affinity of TSL1 toward the investigated metal ions by revealing favorable charge transfer and electron-density distribution around the coordinating sites. During the elution step, protonation of the ligand donor atoms by nitric acid weakens the metal–ligand interactions, resulting in effective desorption of the retained metal ions and regeneration of the sorbent.

### Method Optimization Studies

To ensure high extraction efficiency and robust analytical performance, the key experimental parameters affecting the extraction process were systematically optimized. The investigated variables included sample pH, sorbent amount, extraction time, ligand concentration, and the type and volume of the elution solvent. Optimization was initially carried out using a one-factor-at-a-time (OFAT) strategy, in which one parameter was varied within an appropriate range while all other parameters were kept constant. The influence of each factor was evaluated based on the recovery percentages of the target metal ions. All optimization experiments were performed in at least triplicate, and the resulting recovery data were subjected to one-way analysis of variance (ANOVA) to statistically assess the significance of each parameter. ANOVA was applied solely to determine whether variations in individual factors led to statistically significant differences in extraction efficiency at a 95% confidence level (α = 0.05). Parameters exhibiting significant effects (*p* < 0.05) were further refined, and the optimum conditions were selected according to the highest and most reproducible recovery values. Unlike multivariate experimental designs, such as response surface methodology (RSM), which are primarily used to model interaction effects between variables, the present study focused on independent factor evaluation to achieve reliable and practically applicable optimization within a straightforward experimental framework. This approach enabled clear interpretation of the influence of each parameter and ensured method robustness without introducing unnecessary model complexity. The combined use of OFAT optimization and one-way ANOVA provided both experimental control and statistical validation, strengthening the reliability of the selected extraction conditions.

### Effect of Ligand Concentration

In DmSPE, the performance of the extraction process is dictated by the extent and effectiveness of metal–sorbent interactions, which are governed by the density and distribution of ligand-derived functional groups on the silica surface. Inadequate ligand immobilization yields an insufficient number of accessible coordination sites, thereby limiting complex formation and resulting in low metal retention. In contrast, excessive ligand loading may induce steric congestion and partial pore obstruction, diminishing surface accessibility and hindering the diffusion of metal ions toward active sites.^26^ Consequently, careful optimization of the ligand concentration is essential during sorbent preparation to ensure an optimal balance between surface functionality and accessibility. To investigate the influence of ligand concentration on extraction performance, a series of sorbents was prepared by interacting 10 mL of the Schiff base ligand solutions at concentrations of 5 × 10^–6^, 1 × 10^–5^, 5 × 10^–5^, 1 × 10^–4^, and 2.5 × 10^–4^ mol L^–1^ with 1 g of silica. Subsequently, 30 mg of each prepared sorbent was dispersed into the metal ion solutions and mixed for 10 min to allow sufficient interaction between the sorbent and analytes. After filtration, the residual metal ion concentrations in the filtrates were determined, and the percentage retention of each metal ion was calculated. The experimental results revealed that the sorbent prepared using a ligand concentration of 1 × 10^–4^ mol L^–1^ exhibited the highest overall retention efficiencies for Hg­(II), Pb­(II), Cu­(II), and Cd­(II) ions (Figure 7). At lower ligand concentrations, metal uptake was restricted by the limited availability of coordination sites, whereas higher ligand loadings adversely affected sorption due to steric effects and surface saturation. Accordingly, 1 × 10^–4^ mol L^–1^ was selected as the optimum ligand concentration and employed in all subsequent extraction studies.

**Figure 7:**
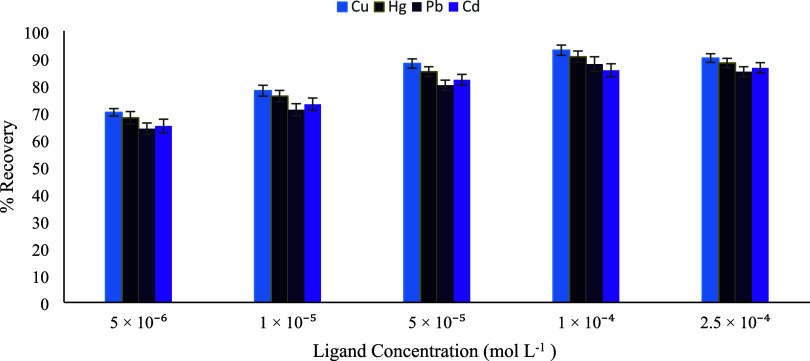
Effect of ligand concentration on the extraction recovery of Hg­(II), Cu­(II), Pb­(II), and Cd­(II) (*n* = 3).

### Optimization of Elution Conditions

In the UA-DmSPE method, efficient desorption of metal ions from the sorbent is a critical step for achieving high extraction recoveries as well as ensuring sorbent reusability. Acidic eluents are commonly preferred for the elution of heavy metals due to their ability to weaken metal–sorbent interactions through protonation of active binding sites. In the present study, nitric acid (HNO_3_) and hydrochloric acid (HCl) were selected as elution solvents since they are the most frequently employed acids in heavy metal microextraction studies and exhibit full compatibility with atomic absorption spectrometry (AAS).^27^ Both acids provide efficient desorption without causing spectral interferences, salt precipitation, or signal instability during AAS measurements. To evaluate the effect of solvent polarity, HNO_3_ and HCl solutions were prepared in both water and methanol and initially tested at a volume of 1 mL. Among the tested eluents, 1.0 mol L^–1^ HNO_3_ prepared in water yielded the highest recovery values for all target metals (Figure 8), demonstrating a stronger elution capability compared to HCl. This behavior can be attributed to the higher oxidative strength of nitric acid, which facilitates the disruption of coordination interactions between metal ions and the functional groups on the sorbent surface. In addition, aqueous HNO_3_ exhibited superior elution performance compared to its methanolic counterpart, indicating that the aqueous medium enhances proton activity and effective metal desorption. The influence of HNO_3_ concentration was further investigated using aqueous solutions, and the highest recoveries were obtained at a concentration of 1.0 mol L^–1^ (Figure 9). At higher acid concentrations, a noticeable decrease in recovery was observed, which may be attributed to excessive proton competition and partial destabilization of desorbed metal species in the eluent phase. Therefore, 1.0 mol L^–1^ HNO_3_ in water was selected as the optimum elution solvent. Optimization of eluent volume is also essential, as lower eluent volumes can provide higher enrichment factors and improved analytical sensitivity. Elution experiments were performed using 0.25–2.0 mL of 1.0 mol L^–1^ HNO_3_ in water. As shown in Figure 10, recoveries for all metals increased with eluent volume up to 0.5 mL, indicating more effective desorption from the sorbent surface. Further increases in eluent volume resulted in decreased recoveries, mainly due to dilution effects rather than insufficient desorption. Consequently, 0.5 mL of 1.0 mol L^–1^ HNO_3_ prepared in water was selected as the optimum eluent volume for subsequent studies.

**Figure 8:**
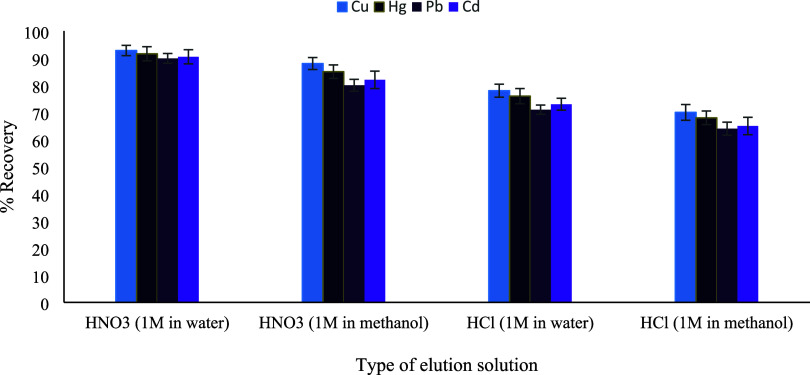
Type of the elution solution (*n* = 3).

**Figure 9:**
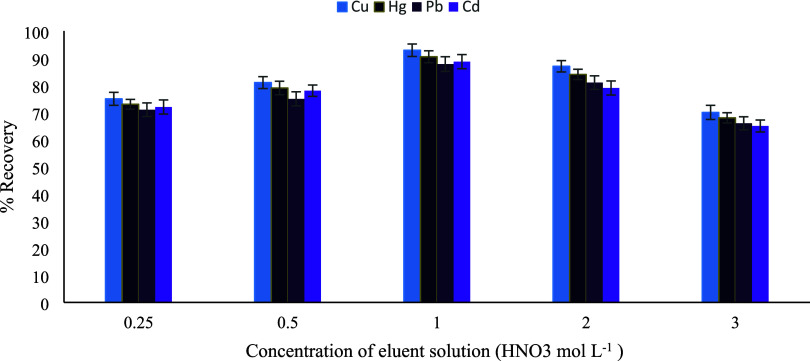
Concentration of the eluent solution (HNO_3_ mol L^–1^) (*n* = 3).

**Figure 10:**
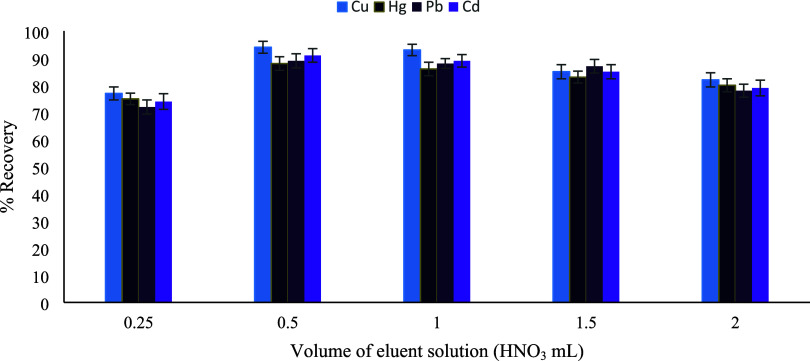
Volume of the eluent solution (HNO_3_ mL) (*n* = 3).

### pH Optimization Studies

In the UA-DMSPE method, the pH of the sample solution plays a crucial role in governing the interaction between metal ions and the functional groups present on the sorbent surface. Therefore, optimization of pH is essential for achieving efficient extraction and reliable analytical performance.^28^ In the present study, the effect of pH on the extraction efficiency of Cu­(II), Hg­(II), Pb­(II), and Cd­(II) ions was investigated over the pH range of 3–9. As illustrated in Figure 11, extraction recoveries for all target metals were strongly dependent on the solution pH. At low pH values (pH 3–4), very low recoveries were obtained, which can be attributed to the excessive presence of hydrogen ions competing with metal ions for the active binding sites on the sorbent surface, thereby suppressing metal adsorption. Upon increasing the pH, a sharp increase in recovery was observed from pH 5, reaching maximum values at pH 6 for all investigated metals. At near-neutral pH, the functional groups on the sorbent surface are sufficiently deprotonated to effectively coordinate metal ions, while the metals remain predominantly in their soluble ionic forms. A slight decrease in recoveries was observed at pH 7, followed by a more pronounced decline at higher pH values. This behavior can be explained by the increased tendency of metal ions, particularly Pb­(II), Cd­(II), and Cu­(II), to form insoluble metal hydroxide species in alkaline media, which reduces their availability for interaction with the sorbent. In addition, Hg­(II) is known to undergo hydrolysis and nonspecific adsorption at elevated pH values, further contributing to the observed decrease in extraction efficiency. Based on these results, pH 6 was selected as the optimum extraction pH for the UA-DMSPE procedure. Although pH 6.0 was found to be optimal for all investigated metals, in Hg­(II)-containing solutions the pH was carefully maintained slightly below 6.0 to avoid hydrolysis and wall adsorption effects.

**Figure 11:**
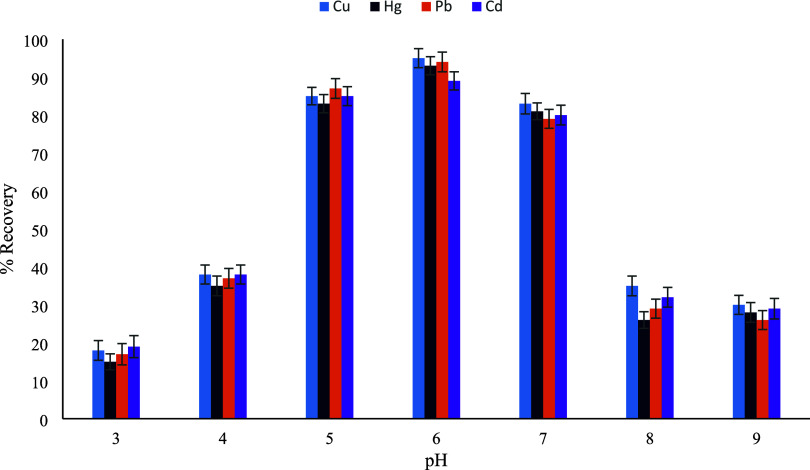
pH optimization (*n* = 3).

### Sorbent Amount Optimization

The amount of sorbent is a critical parameter in dispersive micro-solid-phase extraction, as it directly governs the number of accessible active sites available for metal–ligand interactions.^29^ To evaluate this effect, different amounts of the synthesized sorbent (5–60 mg) were investigated under otherwise constant experimental conditions. As illustrated in Figure 12, the extraction recoveries of Cu­(II), Hg­(II), Pb­(II), and Cd­(II) increased markedly when the sorbent amount was raised from 5 to 40 mg. This behavior can be attributed to the progressive increase in available binding sites, which enhances the probability of complex formation between the metal ions and the functional groups on the sorbent surface. Maximum recoveries (≈90–95%) for all target metal ions were achieved at a sorbent amount of 40 mg, indicating that this quantity provides sufficient surface area and functional group density for efficient sorption. However, further increasing the sorbent amount beyond 40 mg resulted in a noticeable decline in recovery values. This decrease may be associated with particle agglomeration at higher sorbent loadings, which can reduce the effective surface area, as well as with difficulties in the quantitative elution of metal ions from an excessive sorbent mass. Consequently, 40 mg was selected as the optimum sorbent amount for subsequent extraction experiments, ensuring high recovery while maintaining efficient desorption and reproducibility.

**Figure 12:**
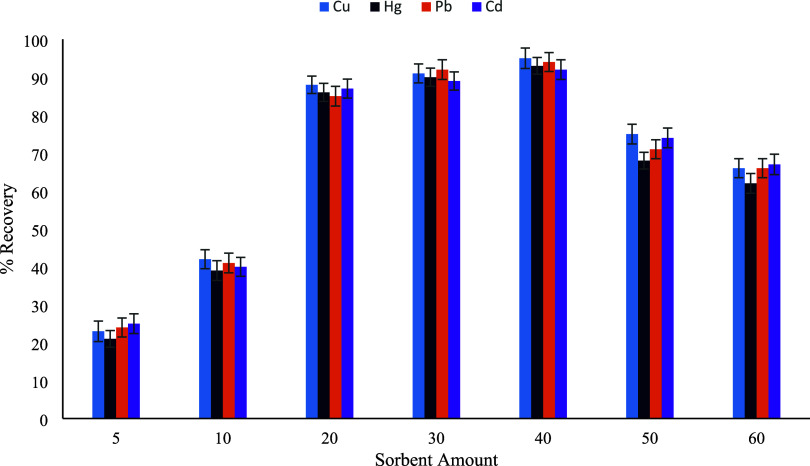
Sorbent amount (*n* = 3).

### Optimization of Extraction Time

Extraction time is a key parameter in ultrasound-assisted dispersive micro-solid-phase extraction, as it directly influences the mass transfer rate between the aqueous phase and the sorbent surface.^30^ The effect of extraction time on the recovery of Cu­(II), Hg­(II), Pb­(II), and Cd­(II) ions was investigated in the range of 5–30 min under otherwise optimized conditions (Figure 13). As can be seen, recoveries increased sharply when the extraction time was extended from 5 to 10 min, indicating that ultrasonic agitation effectively enhances the dispersion of the sorbent and accelerates the interaction between metal ions and the active functional groups. Maximum recoveries, approaching quantitative values (≈95–99%), were obtained at an extraction time of 10 min for all studied metal ions, suggesting that equilibrium between the sorbent surface and the analytes was rapidly established. Prolonging the extraction time beyond this point resulted in a gradual decrease in recovery values. This behavior may be attributed to partial desorption of metal ions back into the aqueous phase or to the weakening of metal–sorbent interactions under prolonged ultrasonic exposure. Similar trends have been reported in previous UA-DMSPE studies using hydrazone- and Schiff base-functionalized silica sorbents, where excessively long sonication times negatively affected extraction efficiency due to disruption of surface complexes. Considering both extraction efficiency and analytical throughput, an extraction time of 10 min was selected as the optimum value for subsequent experiments. This relatively short extraction time highlights the fast kinetics of the proposed method and confirms the beneficial role of ultrasonic assistance in achieving rapid and efficient metal ion extraction.

**Figure 13:**
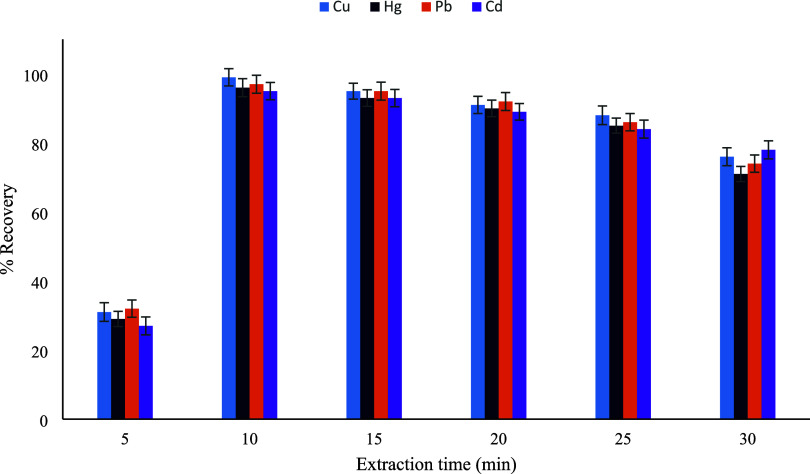
Extraction time (min) (*n* = 3).

The optimum operating conditions of the ultrasound-assisted dispersive micro-solid-phase extraction (UA-DMSPE) method are presented in Table 2. The investigated parameters comprised the ligand concentration employed for silica functionalization, sample pH, sorbent amount, extraction time, and type, concentration, and volume of the elution solvent. Each parameter was systematically optimized using a one-factor-at-a-time (OFAT) strategy, while the remaining variables were kept constant in order to evaluate their individual influence on the extraction efficiency of the target metal ions. The selected optimum conditions were those that provided the highest and most reproducible recovery values for all analytes, ensuring both an efficient extraction performance and methodological robustness.

**Table 2:** Optimized UA-DMSPE Parameters for the Extraction of Hg­(II), Cu­(II), Pb­(II), and Cd­(II)

parameter	optimum condition
Ligand concentration (for sorbent preparation)	1 × 10^–4^ mol L^–1^
Sorbent amount	40 mg
Sample volume	20.0 mL
Sample pH	6.0 (maintained <6.0 for Hg(II))
Extraction technique	Ultrasound-assisted DmSPE
Extraction time	10 min
Eluent type	HNO_3_
Eluent solvent	Water
Eluent concentration	1.0 mol L^–1^ HNO_3_
Eluent volume	0.5 mL

According to the one-way ANOVA results presented in Table 3, all investigated experimental parameters exerted statistically significant effects on the recovery efficiencies of Cu­(II), Cd­(II), Pb­(II), and Hg­(II) ions (*p* < 0.05), confirming the critical role of optimization in the UA-DMSPE procedure. Among the evaluated factors, pH and sorbent amount were identified as the most influential parameters, as evidenced by their consistently higher F-values across all target analytes, indicating a markedly greater between-group variance relative to experimental error. This result clearly demonstrates that solution chemistry and the availability of active binding sites are the dominant controlling factors governing metal–sorbent interactions. In contrast, extraction time exhibited a moderate yet statistically significant effect, suggesting that adsorption equilibrium is rapidly attained within a relatively narrow time window under ultrasonic assistance. Ligand concentration and eluent type displayed comparatively lower *F*-values, implying that once optimal chemical conditions are established, minor variations in these parameters exert a less pronounced influence on overall recovery. Overall, the ANOVA findings statistically validate the robustness of the optimization strategy and demonstrate that the developed method is primarily governed by pH-dependent complexation behavior and sorbent availability rather than by elution efficiency or kinetic factors.

**Table 3:** One-way ANOVA Results for Optimization Parameters Affecting Metal Recoveries

parameter/metal ion	df (T/E)	SS (T/E)	MS (T/E)	*F*-value	*p*-value
pH–Hg(II)	2/6	980.4/15.2	490.2/2.53	193.8	8.2 × 10^–14^
pH–Cu(II)	2/6	905.6/16.0	452.8/2.67	169.6	1.1 × 10^–13^
pH–Pb(II)	2/6	872.3/18.4	436.2/3.07	142.1	2.4 × 10^–12^
pH–Cd(II)	2/6	648.9/19.6	324.5/3.27	99.2	9.3 × 10^–12^
Sorbent amount–Hg(II)	2/6	910.0/14.0	455.0/2.33	195.3	6.5 × 10^–13^
Sorbent amount–Cu(II)	2/6	845.2/13.6	422.6/2.27	186.2	7.4 × 10^–13^
Sorbent amount–Pb(II)	2/6	780.5/16.8	390.3/2.80	139.4	3.1 × 10^–12^
Sorbent amount–Cd(II)	2/6	720.8/18.0	360.4/3.00	120.1	4.2 × 10^–12^
Extraction time–Hg(II)	2/6	640.5/14.6	320.3/2.43	131.8	5.1 × 10^–12^
Extraction time–Cu(II)	2/6	612.4/13.8	306.2/2.30	133.1	4.7 × 10^–12^
Extraction time–Pb(II)	2/6	540.6/15.9	270.3/2.65	102.0	1.6 × 10^–11^
Extraction time–Cd(II)	2/6	580.7/14.7	290.4/2.45	118.6	6.2 × 10^–12^
Ligand concentration–Hg(II)	2/6	410.2/13.2	205.1/2.20	93.2	2.3 × 10^–11^
Ligand concentration–Cu(II)	2/6	445.6/12.4	222.8/2.07	107.6	1.4 × 10^–11^
Ligand concentration–Pb(II)	2/6	380.3/14.8	190.2/2.47	77.0	6.1 × 10^–11^
Ligand concentration–Cd(II)	2/6	355.4/16.2	177.7/2.70	65.8	9.5 × 10^–11^
Eluent type–Hg(II)	2/6	160.4/13.6	80.2/2.27	35.3	8.4 × 10^–6^
Eluent type–Cu(II)	2/6	145.8/12.8	72.9/2.13	34.2	9.1 × 10^–6^
Eluent type–Pb(II)	2/6	128.5/16.5	64.3/2.75	23.4	6.9 × 10^–5^
Eluent type–Cd(II)	2/6	95.6/14.1	47.8/2.35	20.3	8.8 × 10^–5^

### Evaluation of Green Analytical Performance

Considering the increasing emphasis on sustainable analytical methodologies, the environmental performance of the proposed UA-DMSPE method was evaluated by using green analytical metrics to verify its compliance with green chemistry principles.

### Analytical Eco-Scale

The environmental performance of the optimized UA-DMSPE procedure was evaluated using the Analytical Eco-Scale in accordance with the literature-established approach. In this model, penalty points are assigned to factors that adversely affect the environmental friendliness of an analytical method, including reagent hazard, energy consumption, waste generation, and occupational safety. In the present method, the use of a very low volume of aqueous nitric acid for elution (1.0 mL of 1.0 mol L^–1^ HNO_3_) and only dropwise addition of dilute acid/base for pH adjustment resulted in 5 penalty points for reagent hazard. Energy consumption associated with short ultrasound-assisted extraction (10 min), moderate centrifugation steps, and low-energy FAAS detection contributed an additional 5 points. Waste generation was limited to the discarded aqueous sample phase (≈10 mL) and a small volume of acidic eluate after measurement (≈1 mL), yielding only 2 penalty points, while no organic solvent waste was produced.^30^ No additional risks beyond standard laboratory practice were identified, and therefore no penalty points were assigned for occupational safety. Overall, the method accumulated 12 penalty points, corresponding to an Eco-Scale score of 88, which classifies the procedure as an Excellent Green Analysis. The high Eco-Scale score highlights the environmentally benign nature of the developed method, arising from solvent-free operation, minimal reagent and waste volumes, low energy demand, and short analysis time, while maintaining effective analytical performance (Table 4).

**Table 4:** Analytical Eco-Scale Evaluation of the Proposed UA-DmSPE–FAAS Method

category	experimental description (optimized conditions)	penalty points	remarks
Reagent hazard	Elution with 1.0 mL of 1.0 mol L^–1^ HNO_3_ (aqueous). pH adjustment with dilute HNO_3_/NaOH (dropwise). No organic solvent.	5	Low-volume aqueous acid; solvent-free operation
Energy consumption	Ultrasonic extraction (10 min, room temperature); centrifugation (5000 rpm, 10 min × 2); FAAS measurement.	5	Short sonication; moderate centrifugation; low-energy detection (FAAS)
Waste generation	≈10 mL aqueous supernatant discarded per run (sample phase) + 1.0 mL acidic eluate after measurement (total ≈ 11 mL aqueous waste/run). No organic waste.	2	Waste volume mainly equals sample volume; acidic eluate is limited and can be neutralized
Occupational safety	Standard PPE and fume hood for acid handling; no volatile/toxic organic solvents.	0	No additional hazards beyond routine acidic solutions

### Green Analytical Procedure Index (GAPI) Assessment of the Proposed Method

The environmental performance of the optimized UA-DMSPE procedure was evaluated by using the Green Analytical Procedure Index (GAPI). This approach assesses the greenness of an analytical method throughout all stages of the analytical workflow, ranging from sample collection and preparation to instrumental determination and waste management by assigning a three-level color code: green (
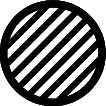
), yellow (
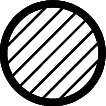
), and red (
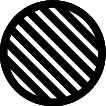
). In this classification, green indicates low environmental impact, yellow represents moderate environmental burden, typically associated with limited use of hazardous reagents or moderate energy demand, while red denotes high environmental impact, usually arising from toxic solvents, large reagent volumes, or energy-intensive instrumentation.^31^ As shown in Table 5, the developed UA-DMSPE method exhibited a predominantly green GAPI profile, with all evaluated steps classified as green (
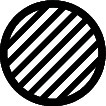
). This result highlights the environmentally benign nature of the entire analytical sequence. The predominance of green zones can be attributed to the use of a small sample volume (10 mL), solvent-free operation, very low reagent consumption (0.5 mL of aqueous nitric acid for elution), simple aqueous-based sample preparation, short ultrasound-assisted extraction time, and the employment of FAAS, which requires a lower energy input compared to plasma-based techniques. In addition, waste generation was restricted to a small volume of neutralizable aqueous waste, and no derivatization or hazardous organic solvents were involved. Overall, the GAPI evaluation clearly demonstrates that the proposed UA-DMSPE procedure achieves excellent environmental compatibility, fully complying with the principles of green analytical chemistry.

**Table 5:**
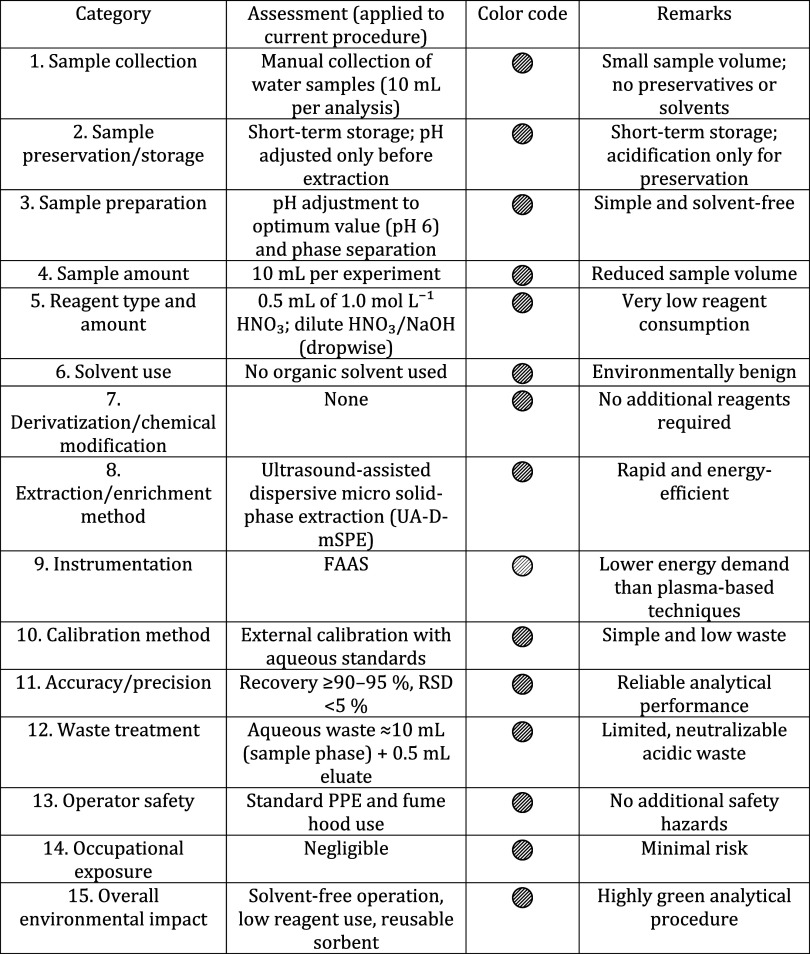
GAPI-Based Greenness Assessment of the Proposed Analytical Method

### AGREE-Based Greenness Evaluation of the Proposed Analytical Method

The greenness of the optimized UA-DMSPE procedure was evaluated using the Analytical GREEnness (AGREE) approach based on the 12 principles of green analytical chemistry. According to the AGREE assessment, the developed method achieved an overall score of 0.85, indicating a high level of environmental sustainability.^32^ High individual scores were obtained for the absence of derivatization (1.00), operator safety (1.00), minimal sample preparation (0.90), waste minimization (0.90), and low reagent and solvent consumption, owing to the use of only 0.5 mL of aqueous nitric acid and the complete avoidance of organic solvents (0.95). The method also benefited from a reduced sample volume (10 mL) and a single-step ultrasound-assisted extraction process, contributing to a high score for procedural simplicity (0.95). Minor score reductions were mainly associated with laboratory-based FAAS detection rather than in situ analysis (0.70), partial manual operation without automation (0.60), and the sequential determination of target metals (0.70) (Table 6). Overall, the AGREE results demonstrate that the proposed analytical method is strongly aligned with the principles of green analytical chemistry, combining an efficient analytical performance with low reagent consumption, limited waste generation, and safe operating conditions.

**Table 6:** AGREE Assessment of the Proposed Analytical Method

No.	green analytical chemistry principle	description (applied to current method)	score (0–1)
1	Direct analytical techniques preferred	Preconcentration (UA-DmSPE) is required prior to FAAS determination.	0.80
2	Minimal sample size and number of steps	10 mL sample; single-step ultrasound-assisted dispersive adsorption and direct acid elution.	0.95
3	In situ measurements if possible	Laboratory-based FAAS; not field-portable.	0.70
4	Minimal sample preparation	Only pH adjustment (optimum pH 6) and phase separation; no digestion for water samples (digestion only for mussel).	0.90
5	Automation and miniaturization	Manual batch/UA-DmSPE operation; not automated.	0.60
6	Avoid derivatization	No derivatization/chemical modification step.	1.00
7	Low reagent and solvent consumption	0.5 mL of 1.0 mol L^–1^ HNO_3_ for elution; no organic solvent used.	0.95
8	Nonhazardous reagents	Mainly aqueous media; dilute acid/base used for pH control; limited chemical hazard.	0.85
9	Energy efficiency	Short sonication (10 min), moderate centrifugation (5000 rpm, 10 min × 2) and FAAS measurement.	0.80
10	Waste minimization and treatment	Aqueous waste ≈10 mL (sample phase) + 0.5 mL eluate per run; neutralizable acidic waste.	0.90
11	Multianalyte or multiparameter capability	Target metals measured by FAAS sequentially (not simultaneous multielement detection).	0.70
12	Operator safety and ergonomic design	Standard PPE and fume hood; no volatile/toxic organic solvents.	1.00

When compared with previously reported microextraction-based methods for the determination of heavy metals, such as DLLME, IL-DLLME, CPE, and conventional SPE, the proposed UA-DMSPE approach demonstrates a clear environmental advantage. Most reported methods involve the use of organic solvents, larger reagent volumes, or longer extraction times, which increases their ecological footprint. In contrast, the present method operates without organic solvents, requires only a small volume of aqueous nitric acid for elution, and employs a short ultrasound-assisted extraction step. These features result in lower waste generation, reduced reagent consumption, and improved energy efficiency, which are reflected in the high Eco-Scale score, predominantly green GAPI profile, and high AGREE value obtained in this study.^33^

### Interference Studies

The selectivity of the proposed UA-DMSPE method was evaluated by investigating the influence of various potentially interfering ions commonly encountered in environmental and marine matrices. For this purpose, representative alkali and alkaline earth metals (Na^+^, K^+^, Mg^2+^, Ca^2+^), transition metals (Fe^3+^, Al^3+^, Mn^2+^, Cr^3+^, Zn^2+^, Co^2+^, Ni^2+^, and Pb^2+^), and major inorganic anions (Cl^–^, NO_3_^–^, SO_4_^2–^, PO_4_^3–^, and HCO_3_^–^) were individually introduced into 10 mL solutions containing the target analytes under the optimized extraction conditions. The tolerance limit was defined as the maximum concentration of the interfering ion causing less than 5% deviation in analyte recovery. As summarized in Table 7, the proposed method exhibited a high tolerance toward major matrix constituents. Alkali and alkaline earth ions, as well as common anions, showed negligible interference even at relatively high concentrations (up to 1000 μg mL^–1^), with recovery values remaining above 96% for Hg­(II), Cu­(II), Pb­(II), and Cd­(II). This behavior indicates that nonspecific electrostatic interactions play a minor role in the extraction process, and that the sorption mechanism is predominantly governed by selective coordination between the metal ions and the functional groups on the silica surface. Transition metal ions displayed slightly lower tolerance limits, particularly for Fe^3+^, Zn^2+^, Ni^2+^, and Pb^2+^, which can be attributed to competitive complexation with similar coordination preferences. Nevertheless, even under these more challenging conditions, the recoveries of the target analytes remained within an acceptable range (94.8–97.9%), with relative standard deviations generally below ±4%. Such behavior is consistent with previously reported UA-DMSPE systems employing hydrazone- and Schiff base-functionalized silica sorbents, where competitive interactions with multivalent metal ions resulted in marginal but tolerable decreases in extraction efficiency. Overall, the interference study confirms that the developed method possesses strong selectivity and robustness against a wide range of coexisting ions. The ability to maintain high and reproducible recoveries in the presence of both major inorganic constituents and potentially competing transition metals demonstrates the suitability of the proposed approach for the determination of Hg­(II), Cu­(II), Pb­(II), and Cd­(II) in complex environmental and marine samples.

**Table 7:** Interference Effect on the Extraction of Studied Ions (*n* = 3)

interfering ion	source salt	tolerance limit (μg mL^–1^)	Hg(II) Rec. (%)	Cu(II) Rec. (%)	Pb(II) Rec. (%)	Cd(II) Rec. (%)
Na^+^	NaCl	1000	96.8 ± 2.4	97.2 ± 1.9	96.1 ± 2.7	97.5 ± 1.8
K^+^	KCl	1000	96.1 ± 2.7	96.8 ± 2.3	95.7 ± 3.1	96.9 ± 2.0
Mg^2+^	Mg(NO_3_)_2_	500	97.3 ± 1.8	96.5 ± 2.1	95.9 ± 2.6	96.7 ± 1.9
Ca^2+^	CaCl_2_	500	96.4 ± 2.2	97.1 ± 1.7	96.0 ± 2.4	96.8 ± 2.1
Fe^3+^	FeCl_3_	80	95.2 ± 3.6	95.7 ± 3.1	94.9 ± 3.8	95.6 ± 3.0
Al^3+^	Al(NO_3_)_3_	50	95.8 ± 2.9	96.3 ± 2.7	95.1 ± 3.4	96.0 ± 2.6
Mn^2+^	MnCl_2_	100	96.0 ± 2.1	96.7 ± 2.4	95.6 ± 2.8	96.4 ± 2.2
Cr^3+^	Cr(NO_3_)_3_	100	95.4 ± 3.2	95.9 ± 3.0	95.0 ± 3.5	95.8 ± 2.9
Zn^2+^	Zn(NO_3_)_2_	50	95.9 ± 2.6	96.8 ± 2.9	95.4 ± 3.7	97.1 ± 2.4
Co^2+^	Co(NO_3_)_2_	80	95.6 ± 3.0	95.9 ± 2.8	94.8 ± 3.4	96.2 ± 2.7
Ni^2+^	NiCl_2_	90	95.7 ± 2.5	96.4 ± 2.6	95.2 ± 3.1	96.6 ± 2.3
Pb^2+^	Pb(NO_3_)_2_	50	95.3 ± 3.4	95.6 ± 3.2		95.9 ± 3.0
Cl^–^	NaCl	1000	96.5 ± 2.1	97.0 ± 2.0	96.3 ± 2.5	97.2 ± 1.9
NO_3_^–^	NaNO_3_	1000	96.9 ± 1.7	97.4 ± 1.6	96.8 ± 2.0	97.6 ± 1.5
SO_4_^2–^	Na_2_SO_4_	1000	97.1 ± 1.9	97.8 ± 1.4	97.0 ± 1.8	97.9 ± 1.3
PO_4_^3–^	Na_3_PO_4_	1000	96.2 ± 2.3	96.7 ± 2.1	96.0 ± 2.6	96.8 ± 2.0
HCO_3_^–^	NaHCO_3_	1000	96.6 ± 2.0	97.1 ± 1.8	96.4 ± 2.2	97.0 ± 1.7

### Effect of Sample Volume

The effect of sample volume on extraction efficiency was systematically investigated over the range of 5–25 mL. As illustrated in Figure 14, quantitative recoveries were maintained for all target metal ions as the sample volume increased up to 20 mL, with maximum recoveries (≥94%) observed at this volume. This behavior indicates efficient interaction between the metal ions and the available active binding sites of the sorbent. Given the fixed elution volume of 0.5 mL, increasing the sample volume to 20 mL led to a substantial enhancement in the preconcentration factor (approximately 40-fold) without any loss in extraction efficiency.^34^ In contrast, a decline in recovery was observed at 25 mL, which can be attributed to saturation of the sorbent’s binding sites and limited sorbent capacity, despite the theoretically higher enrichment factor. Accordingly, a sample volume of 20 mL was selected as the optimum condition, providing an optimal compromise between high recovery, sorbent capacity, and preconcentration efficiency. During the preliminary stage of method development, an initial sample volume of 10 mL was employed for the optimization of other experimental parameters to ensure rapid screening and method stability. Following the evaluation of sample volume, the optimized value of 20 mL was adopted in all subsequent experiments, including method validation and real sample analyses.

**Figure 14:**
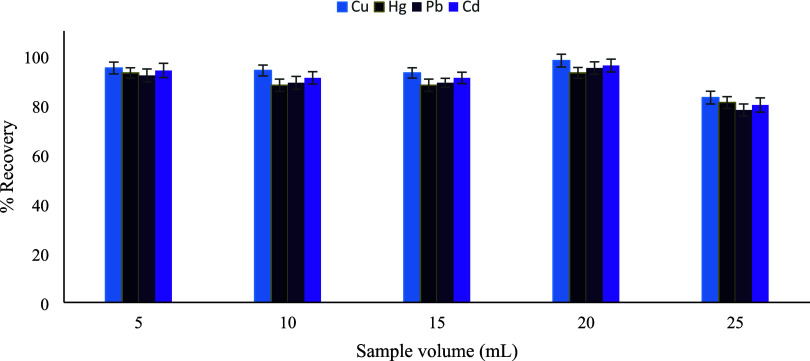
Effect of the sample volume (*n* = 3).

### Evaluation of Sorbent Reusability in Repeated Extraction Cycles

The reusability of the sorbent was evaluated through five consecutive adsorption–desorption cycles under the optimized UA-DMSPE conditions for Cu­(II), Hg­(II), Pb­(II), and Cd­(II) ions. In the first extraction cycle, high recovery values were obtained (98% for Cu­(II), 93% for Hg­(II), 95% for Pb­(II), and 96% for Cd­(II)), demonstrating the strong affinity of the sorbent toward the target metal ions. With increasing reuse cycles, a gradual decrease in recovery values was observed for all metals. After the third cycle, the reduction in recovery remained limited to approximately 5–6%, indicating that more than 94% of the initial extraction efficiency was preserved. Such minor losses are commonly reported for UA-DmSPE sorbents and are considered acceptable in terms of sorbent stability and practical applicability. The observed decrease can be attributed to partial loss of active binding sites and incomplete regeneration during repeated acid elution steps. A more pronounced decline in recovery was detected after the fourth and fifth cycles, with recovery values decreasing to 87–90% for Cu­(II), 85–88% for Hg­(II) and Cd­(II), and approximately 83–86% for Pb­(II) (Figure 15). Based on these results, three reuse cycles were selected as the optimum operational limit for the sorbent, providing a reliable compromise between analytical performance and sorbent durability.

**Figure 15:**
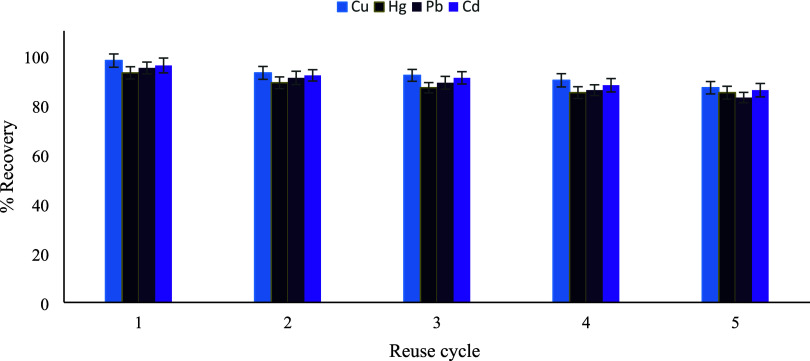
Reusability performance of the TSL1-functionalized silica sorbent for Hg­(II), Cu­(II), Pb­(II), and Cd­(II) over five consecutive adsorption–desorption cycles (*n* = 3).Method validation.

The analytical performance of the proposed UA-DMSPE–FAAS method was evaluated in terms of linearity, sensitivity, and precision for Cu­(II), Hg­(II), Pb­(II), and Cd­(II) ions. As summarized in Table 8, good linear relationships were obtained over the concentration ranges of 2–500 μg L^–1^ for Cu­(II), 5–500 μg L^–1^ for Hg­(II), 3–500 μg L^–1^ for Pb­(II), and 5–500 μg L^–1^ for Cd­(II), with correlation coefficients (R^2^) higher than 0.993 for all analytes, indicating satisfactory linearity of the method. Method sensitivity was assessed by determining the limits of detection (LOD) and limits of quantification (LOQ). The LOD values were found to be 0.60 μg L^–1^ for Cu­(II), 1.50 μg L^–1^ for Hg­(II), 0.90 μg L^–1^ for Pb­(II), and 1.50 μg L^–1^ for Cd­(II). The corresponding LOQ values were 2.00, 5.00, 3.00, and 5.00 μg L^–1^, respectively. The LOQ values were approximately 3.3 times higher than the corresponding LODs, confirming the reliability of the signal-to-noise-based calculations. Precision was evaluated in terms of intraday and interday repeatability at two concentration levels (10 and 50 μg L^–1^). The intraday precision, expressed as relative standard deviation (%RSD), ranged between 1.8–2.4% at 10 μg L^–1^ and 2.2–3.0% at 50 μg L^–1^. Similarly, interday %RSD values were within the range of 2.1–2.9%, demonstrating good repeatability and reproducibility of the proposed method. Overall, these validation results confirm that the developed UA-DMSPE method provides adequate sensitivity, excellent linearity, and satisfactory precision for the determination of trace-level Cu­(II), Hg­(II), Pb­(II), and Cd­(II) ions.

**Table 8:** Validation Parameters of the Proposed UA-DMSPE–FAAS Method for Hg­(II), Cu­(II), Pb­(II), and Cd­(II)

validation parameter	Cu(II)	Hg(II)	Pb(II)	Cd(II)
Linear range (μg L^–1^)	2–500	5–500	3–500	5–500
*R* ^2^ (*n* = 5)	0.9965	0.9941	0.9953	0.9937
LOD (μg L^–1^, *n* = 10)	0.60	1.50	0.90	1.50
LOQ (μg L^–1^, *n* = 10)	2.00	5.00	3.00	5.00
Intraday precision (10 μg L^–1^, %RSD, *n* = 7)	1.8	2.2	2.0	2.4
Intraday precision (50 μg L^–1^, %RSD, *n* = 7)	2.5	2.9	2.2	3.0
Interday precision (10 μg L^–1^, %RSD, *n* = 7)	2.6	2.4	2.9	2.1
Interday precision (50 μg L^–1^, %RSD, *n* = 7)	1.9	2.2	2.1	2.5

### Application to Real Samples and Evaluation of the Matrix Effect

The evaluation of matrix effects is a critical step in assessing the reliability and practical applicability of analytical methods, particularly when complex environmental and biological matrices are involved. Seawater and mussel samples contain high concentrations of dissolved salts, organic matter, and biomolecules, which may interfere with metal extraction and detection by causing signal suppression or enhancement. Therefore, the matrix effect of the proposed UA-DMSPE method was systematically investigated using seawater and mussel samples spiked with Hg­(II), Pb­(II), Cd­(II), and Cu­(II) at two concentration levels (20 and 50 μg L^–1^). As summarized in Table 9, moderate signal suppression was observed for all target metal ions in both matrices, as indicated by negative percentage differences (%Diff). In seawater samples, %Diff values ranged from 8.33% to 11.91%, reflecting the influence of high ionic strength and competing cations on metal sorbent interactions. Nevertheless, recovery values remained consistently high (92.2–96.3%), demonstrating that the developed method effectively compensates for salinity-related matrix effects. In mussel samples, slightly higher signal suppression was recorded (%Diff between 10.61% and 13.60%), which can be attributed to the complex organic composition of the biological matrix, including proteins and lipid fractions capable of interacting with metal ions. Despite this increased matrix complexity, recovery values remained within acceptable limits (90.2–94.8%) for all analytes, confirming that the extraction efficiency of the sorbent was not significantly compromised. The relatively higher standard deviations observed for mussel samples further support the expected heterogeneity of biological matrices rather than methodological instability. Overall, the obtained matrix effect values did not exceed the generally accepted ±15% threshold, indicating that matrix-induced interferences were effectively controlled. These findings confirm that the proposed UA-DMSPE method exhibits strong matrix tolerance and provides reliable and accurate quantification of Hg­(II), Pb­(II), Cd­(II), and Cu­(II) ions in both saline and biological real samples. The results clearly demonstrate the suitability of the developed method for routine application in complex environmental and seafood-related matrices.

**Table 9:** Recovery and Matrix Effect Evaluation of Hg­(II), Cu­(II), Pb­(II), and Cd­(II) in Seawater and Mussel Samples (*n* = 3)

matrix	metal ion	spiked (μg L^–1^)	measured (μg L^–1^ ± SD)	recovery (%)	matrix effect (μg L^–1^ ± SD)	% Diff
Seawater	Hg(II)	20	18.90 ± 1.10	94.50	21.05 ± 1.08	–10.21%
	Hg(II)	50	46.60 ± 1.25	93.20	52.90 ± 1.32	–11.91%
	Pb(II)	20	19.10 ± 0.98	95.50	21.10 ± 1.02	–9.48%
	Pb(II)	50	47.20 ± 1.18	94.40	52.80 ± 1.24	–10.61%
	Cd(II)	20	18.80 ± 1.02	94.00	20.95 ± 1.06	–10.26%
	Cd(II)	50	46.10 ± 1.22	92.20	51.70 ± 1.28	–10.83%
	Cu(II)	20	19.25 ± 0.95	96.25	21.00 ± 1.00	–8.33%
	Cu(II)	50	47.10 ± 1.15	94.20	52.30 ± 1.20	–9.94%
Mussel	Hg(II)	20	18.35 ± 1.55	91.75	21.10 ± 1.60	–13.03%
	Hg(II)	50	45.10 ± 1.82	90.20	52.20 ± 1.90	–13.60%
	Pb(II)	20	18.70 ± 1.40	93.50	21.40 ± 1.45	–12.62%
	Pb(II)	50	45.85 ± 1.76	91.70	52.70 ± 1.84	–13.00%
	Cd(II)	20	18.90 ± 1.32	94.50	21.40 ± 1.36	–11.68%
	Cd(II)	50	46.15 ± 1.70	92.30	52.80 ± 1.78	–12.59%
	Cu(II)	20	18.95 ± 1.28	94.75	21.20 ± 1.34	–10.61%
	Cu(II)	50	46.40 ± 1.62	92.80	52.60 ± 1.70	–11.79%

### Comparison of the Suggested Method with Other Methods

As summarized in Table 10, most of the previously reported microextraction and preconcentration methods for heavy metal determination have been primarily applied to aqueous matrices, such as tap water, river water, or groundwater. Although satisfactory analytical performances have been reported for these relatively simple matrices, the applicability of many methods to complex environmental or biological samples remains limited. In contrast, the proposed UA-DMSPE method was successfully applied to highly complex matrices, namely seawater and mussel samples, where strong matrix effects are commonly expected due to high salinity, organic content, and coexisting ions. Despite these challenges, the developed method provided high and reproducible recoveries (90.2–96.3%) for all target metal ions, demonstrating its robustness and matrix tolerance. Compared with previously reported FAAS-based methods, the present approach offers comparable or wider linear ranges (up to 500 μg L^–1^) and competitive detection limits (0.60–1.50 μg L^–1^), while maintaining a preconcentration factor of 40 with a simple and rapid experimental procedure. Although some literature methods report higher enrichment factors, these often rely on more complex extraction schemes or advanced instrumentation. Overall, the key advantage of the proposed method lies in its successful performance in complex real samples, rather than being limited to aqueous matrices. The combination of satisfactory sensitivity, moderate enrichment, and applicability to seawater and biological samples highlights the practical utility of the developed UA-DMSPE method for routine multimetal analysis in challenging matrices.

**Table 10:** Comparison of the Developed DmSPE Method with Other Extraction Methods in the Literature

method	detection	metal	linear range (μg/L)	LOD (μg/L)	LOQ (μg/L)	preconcentration Factor	real samples	recovery (%)	reference
UA-DMSPE	FAAS	Cu(II), Hg(II), Pb(II), Cd(II)	Cu(II): 2–500	0.60–1.50	2.00–5.00	40	Seawater and mussels	90.2–96.3	This study
			Hg(II): 5–500						
			Pb(II): 3–500						
			Cd(II): 5–500						
UA-DMSPE	ICP-OES	Pb(II)	Pb(II): 0.2–350	0.037–0.054	0.12–0.18	4	Tap water and River water	98.8–106	^34^
		Cd(II)	Cd(II): 0.1–400						
UA-DMSPE	FAAS	Ni(II)		10–20	30–60	37	Soil and Lake sediment	90–117	^35^
		In(III)							
UA-DMSPE	CV-AAS	Hg(II)		0.004	0.013	35	Lake water and River water	97–118	^36^
UA-DMSPE	FAAS	Co(II)	Co(II): 1–300	0.3–0.6	1.0–2.0	20	Tap water and River water	94–103	^37^
		Ni(II)	Ni(II): 2–400						
UA-DMSPE	FAAS	Cd(II)	Cd(II): 5–3000	1.8	6.0	4	wastewater	90.2–96.3	^38^
UA-DMSPE	FI-HG-AAS	As(III)	As(III): 1–40	0.3–0.5	1.0–1.7	10	Groundwater, River water and Tap water	92–105	^39^
		As(V)	As(V): 2–50						
UA-DMSPE	FAAS	Cu(II)	Cu(II): 0.3–350	0.29–0.77	0.26–0.98	100	Honey	92–104	^40^
		Cd(II)	Cd(II): 0.5–225						
		Pb(II)	Pb(II): 1–400						
UA-DMSPE	ICP-MS	Cd(II)	Cd(II), Co(II), Cu(II), Ni(II), Pb(II), Zn(II): 0.1–500	0.03–0.112	0.1–3.75	10	Well water, treated well water, and river water	96.2–103.6	^41^
		Co(II)							
		Cu(II)							
		Ni(II)							
		Pb(II)							
		Zn(II)							

### Molecular Docking Results

Figure 16 illustrates the binding pockets associated with the most favorable docking scores, highlighting the interaction patterns formed between each protein target and its corresponding ligand. In addition, Figure 16 presents two-dimensional interaction maps that provide a detailed depiction of ligand binding orientations as well as the key amino acid residues involved in stabilizing the protein–ligand complexes. The results of these studies for TSL1 compound are presented in Table 11.

**Figure 16:**
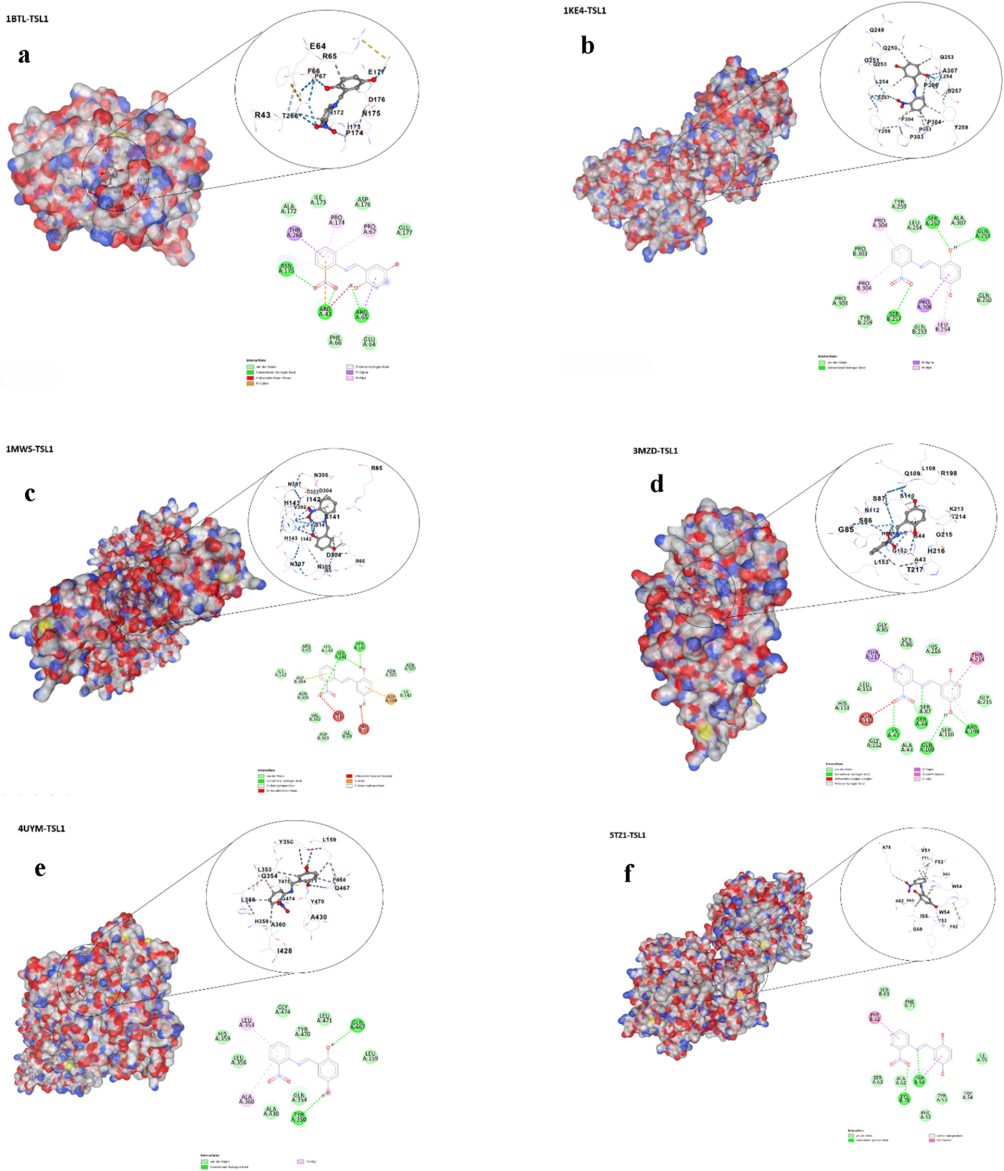
Molecular docking representations of the highest binding affinity cavities between compound TSL1 and selected target proteins: (a) 1BTL, (b) 1KE4, (c) 1MWS, (d) 3MZD, (e) 4UYM, and (f) 5TZ1. For each protein, the three-dimensional surface view of the binding cavity and the corresponding two-dimensional interaction diagram illustrating key ligand–residue interactions are presented.

**Table 11:** Molecular Docking Results of TSL1 with Selected Target Proteins (1BTL, 1KE4, 1MWS, 3MZD, 4UYM, and 5TZ1), Including the Highest Binding Affinity Values and Key Interaction Parameters

receptor (protein)	Cur Pocket ID	Vina Score (kcal/mol)	cavity volume (Å^3^)	center (*x*, *y*, *z*)	docking size (*x*, *y*, *z*)	contact residues
1BTL	C2	–7.3	254	22, −1, 30	20, 20, 20	**Chain A:** ARG43 GLU63 GLU64 ARG65 PHE66 PRO67 ALA172 ILE173 PRO174 ASN175 ASP176 GLU177 ARG178 THR180 ARG241 SER243 THR266 GLY267
1KE4	C1	–8.2	3505	52, −2, 23	26, 32, 35	**Chain A:** LEU85 ASN237 LEU238 LYS239 PRO240 LEU241 ASP242 GLN250 GLN253 LEU254 GLN256 SER257 TYR259 PRO303 PRO304 THR305 PRO306 ALA307 ARG309 PHE328 PRO330 GLU333
						**Chain B:** LEU85 LYS246 THR247 GLN249 GLN250 GLY251 GLN253 LEU254 SER257 TYR259 PRO303 PRO304 THR305 PRO306 ALA307
1MWS	C5	–8.2	949	17, 38, 8	20, 20, 20	**Chain A:** GLU61 ARG65 LYS68 ILE69 GLN140 SER141 ILE142 HIS143 VAL302 ASP303 ASP304 ASN305 ASN307
						**Chain B:** GLU61 ARG65 ILE69 GLN140 SER141 ILE142 HIS143 VAL302 ASP303 ASP304 ASN305 SER306 ASN307
3MZD	C1	–7.1	965	45, 6, 28	20, 26, 20	**Chain A:** ALA43 SER44 LYS47 LYS84 GLY85 SER86 SER87 LEU108 GLN109 SER110 ASN112 HIS151 GLY152 LEU153 ASP154 ARG198 ASN199 GLY200 LEU201 LYS213 THR214 GLY215 HIS216 THR217 GLY221 TYR222 ASN223 LEU224 PHE245 ARG248 GLU249
4UYM	C3	–7.9	2737	123, 195, −10	20, 30, 26	**Chain A:** LYS151 LEU154 LEU159 TYR350 LEU353 GLN354 LYS355 LEU356 ASP357 HIS359 ALA360 ILE363 ILE428 ALA430 SER431 ALA432 LYS437 LEU444 VAL445 SER446 ARG462 CYS463 ILE464 GLY465 GLU466 GLN467 PHE468 TYR470 LEU471 GLN472 GLY474 THR475
5TZ1	C3	–8.0	1811	75, 51, 24	20, 20, 20	**Chain A:** VAL51 PHE52 TYR53 TRP54 ILE55 PHE58 GLY59 SER60 ALA61 ALA62 SER63 GLN66 GLU70 PHE71 SER74 CYS75 LYS78
						**Chain B:** VAL51 PHE52 TRP54 ILE55 SER60 ALA62 SER63 PHE71 SER74 LYS78

The molecular docking heatmap of the protein–ligand interactions, where darker colors represent lower binding energies and thus stronger binding affinities, is shown in Table 12.

**Table 12:**
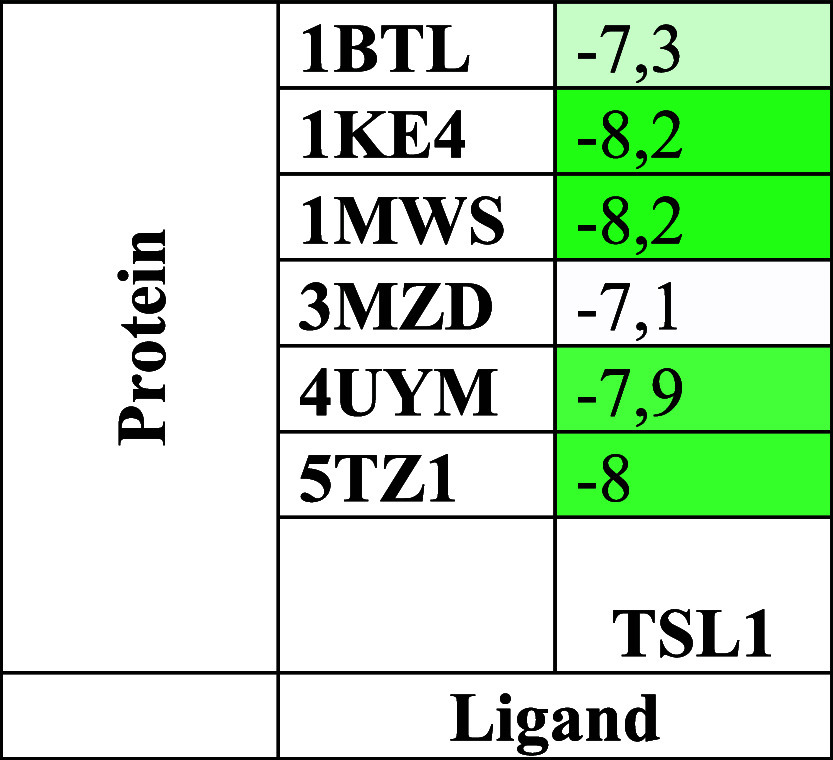
Molecular Docking Heatmap of Protein and Ligands (Binding Affinities Are Displayed by Color)^a^

a The darker color means lower binding energy, which indicates better binding.

Molecular docking calculations conducted using CB-Dock2 indicated that TSL1 binds favorably to all six protein targets, with binding energies spanning from −7.1 to −8.2 kcal·mol^–1^ and accommodating cavities of markedly different volumes (254–3505 Å^3^). For TEM-1 β-lactamase (1BTL), TSL1 was positioned within the compact C2 pocket (254 Å^3^) with a Vina score of −7.3 kcal·mol^–1^, and the 2D interaction map suggests stabilization through two conventional hydrogen bonds (notably involving ASN175 and ARG65), together with an electrostatic/aromatic contribution via a π–cation contact with ARG43 and additional hydrophobic reinforcement through π–alkyl interactions with PRO67 and PRO174; a localized π–sigma contact (THR266) is also observed, while a single unfavorable donor–donor contact around the ARG43 region implies a partially strained polar alignment that may limit further affinity gains in this tight cavity.^42^ In contrast, AmpC β-lactamase (1KE4) produced one of the strongest affinities for TSL1 (−8.2 kcal·mol^–1^) within the large C1 cavity (3505 Å^3^), where the pose is supported by two conventional hydrogen bonds (with GLN253 and SER257) and multiple hydrophobic/aromatic contacts dominated by π–alkyl interactions involving PRO304/PRO306 (with an accompanying π–sigma component around PRO306), consistent with a stable orientation reinforced by a Pro-rich microenvironment.^43^ Docking to PBP2a (1MWS) likewise yielded a favorable score (−8.2 kcal·mol^–1^) in the C5 cavity (949 Å^3^), and the interaction diagram indicates two conventional hydrogen bonds mediated by SER141 (chains A/B) coupled with π–anion contacts to ASP304 and ASP142, supporting a binding mode governed by cooperative polar anchoring and electrostatic complementarity; however, the presence of an unfavorable contact involving HIS143 (donor/acceptor mismatch) suggests that the pose may be energetically optimized primarily by the Ser/Asp network rather than by idealized histidine engagement.^44^ For PBP5 (3MZD), TSL1 exhibited the weakest (yet still favorable) score (−7.1 kcal·mol^–1^) within the C1 pocket (965 Å^3^), where the 2D map highlights a predominantly polar stabilization pattern: conventional hydrogen bonds are apparent (e.g., LYS47 and ARG198, with additional Ser-mediated contacts such as SER44/SER110), while aromatic reinforcement is comparatively modest and occurs mainly through π–sigma (THR217) and amide−π/π–alkyl contributions (THR214); notably, an unfavorable acceptor–acceptor contact with ASN112 is present, which may rationalize the slightly reduced affinity relative to the other targets.^45^ For CYP51B (4UYM), TSL1 bound in the C3 cavity (2737 Å^3^) with −7.9 kcal·mol^–1^, and the interaction profile is comparatively “clean,” being driven by two conventional hydrogen bonds (to GLN467 and TYR350) accompanied by π–alkyl contacts with LEU353 and ALA360, reflecting balanced polar anchoring plus hydrophobic packing within the P450 channel. Similarly, docking to CYP51 (5TZ1) returned −8.0 kcal·mol^–1^ in the C3 cavity (1811 Å^3^), where the ligand is supported by a mixed interaction pattern comprising a conventional hydrogen bond with LYS78, a carbon–hydrogen bond involving TRP54, and prominent aromatic reinforcement via π–π stacking (notably with PHE52 and TRP54), consistent with strong π-surface complementarity in the CYP51 binding channel.^46^ Collectively, these fingerprints show that TSL1 can adapt to diverse binding-site chemistries by switching between Ser/Gln-driven hydrogen-bond anchoring (1KE4/1MWS/4UYM), Pro- and aromatic-assisted hydrophobic/π stabilization (1KE4/5TZ1), and mixed polar−π engagement in constrained pockets (1BTL/3MZD), which plausibly underpins its reproducibly favorable docking behavior across both β-lactam–associated bacterial enzymes and CYP51 targets ligand systems.

### Density Functional Theory (DFT) Results

The optimized geometries of TSL1 and its metal complexes are presented in Figure 17.

**Figure 17:**
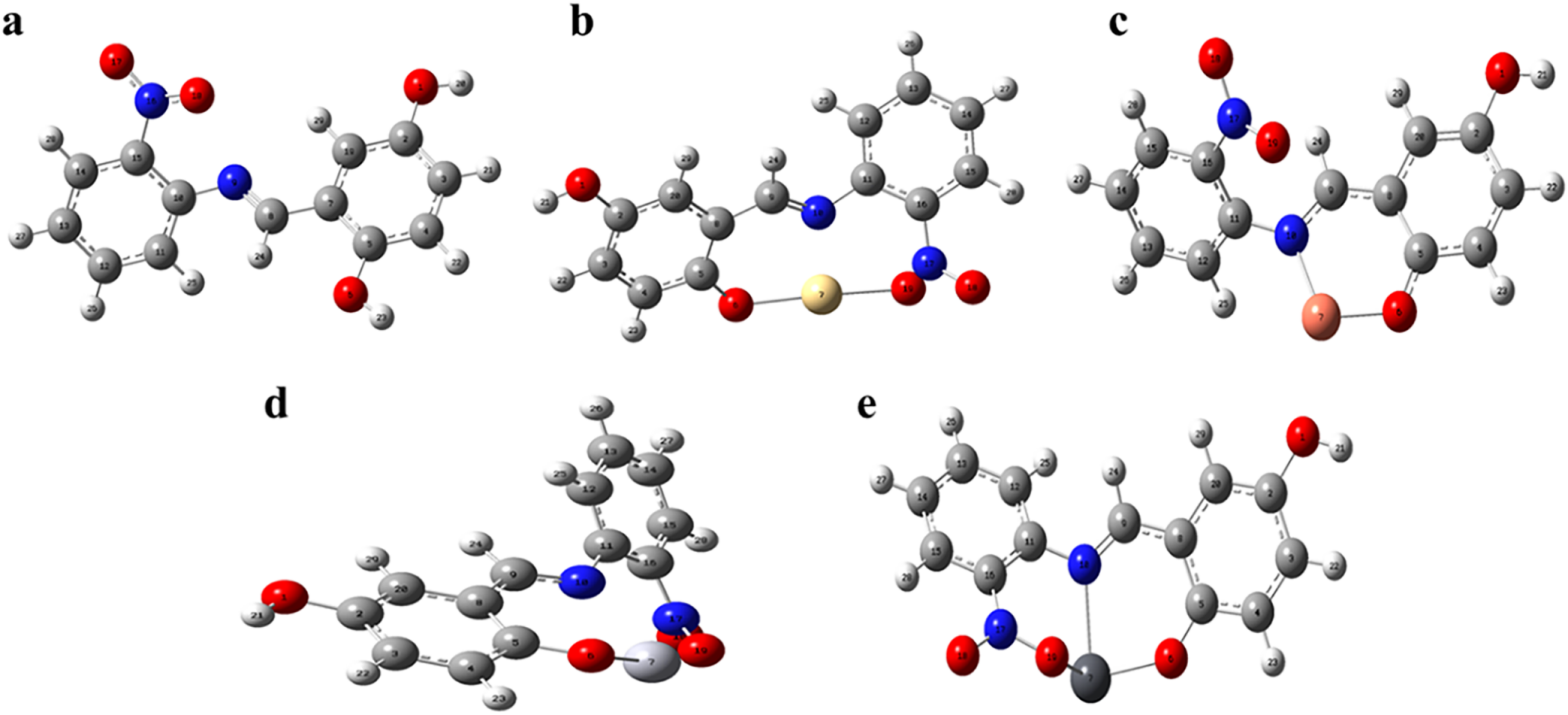
Optimized geometries of (a) TSL1, (b) TSL1-Cd, (c) TSL1-Cu, (d) TSL1-Hg, and (e) TSL1-Pb.

The molecular electrostatic potential (MEP) surfaces of TSL1 and its Cd­(II), Cu­(II), Hg­(II), and Pb­(II) complexes (Figure 18) clearly illustrate how metal coordination reshapes the electrostatic landscape by altering the balance between electron-rich (negative) and electron-deficient (positive) regions across the ligand framework. In the free ligand TSL1 (Figure 18a), the most negative potential (red) is concentrated around the heteroatom-rich segment (primarily the coordinating N/O/S-containing region), identifying these atoms as the dominant nucleophilic centers that are predisposed to metal binding. The positive potential (blue) is mainly distributed over peripheral hydrogen-rich/aromatic regions, consistent with an intrinsic donor–acceptor separation within the uncoordinated ligand. Upon complex formation, the emergence of an intense positive domain (blue) in the vicinity of the metal center becomes evident, reflecting the electrophilic character of the coordinated cation and the polarization induced by ligand-to-metal electron donation. For TSL1–Cd (Figure 18b), the surface shows a moderate and relatively balanced polarization: negative regions remain observable around the donor atoms, while a distinct but not excessively dominant positive region develops near the coordination site. This pattern is consistent with a stable complex in which charge redistribution is appreciable but not extreme. In contrast, the Cu­(II) complex (TSL1–Cu), Figure 18c displays a much more pronounced positive potential (a large, intense blue domain) localized around the metal-coordination region, accompanied by comparatively localized negative patches around the donor atoms. This strong electrostatic polarization indicates a more substantial metal-induced perturbation of the ligand’s electron density and suggests an enhanced tendency toward charge separation/redistribution, in line with stronger electronic coupling within the coordination environment. A similar yet even more spatially extended positive region is observed for the Hg­(II) complex (TSL1–Hg, Figure 18d), where the blue domain spreads broadly over the molecular surface. The extensive positive potential around the coordination zone implies marked electrophilic character and strong polarization effects, consistent with significant electron-density withdrawal from the ligand upon binding to the soft Hg­(II) center. Notably, the Pb­(II) complex (TSL1–Pb, Figure 18e) appears largely uniform and weakly polarized on the displayed surface, with the electrostatic potential dominated by green regions and only limited, less distinct red/blue separation. Based on the visual distribution in this figure, Pb­(II) coordination produces a more electrostatically screened/delocalized surface pattern compared with Cu­(II) and Hg­(II), suggesting that the nucleophilic/electrophilic domains are less sharply differentiated in the plotted MEP representation. Overall, the MEP maps indicate that coordination generally shifts electron density toward the donor atoms (negative regions) while generating a metal-centered positive domain, with the most pronounced polarization observed for the Cu­(II) and Hg­(II) complexes, a moderate pattern for Cd­(II), and a more uniform/less contrasted surface for Pb­(II) in the presented plots. This comparative electrostatic behavior supports the conclusion that the identity of the metal center critically governs the degree of charge redistribution and the resulting interaction potential of the complexes.^47^ DFT calculations were performed using a simplified 1:1 metal–ligand model to provide a theoretical perspective on possible coordination interactions. This study does not rely on the isolation of separate metal complexes; therefore, the optimized structures represent theoretical interaction models rather than experimentally validated stoichiometric complexes.

**Figure 18:**
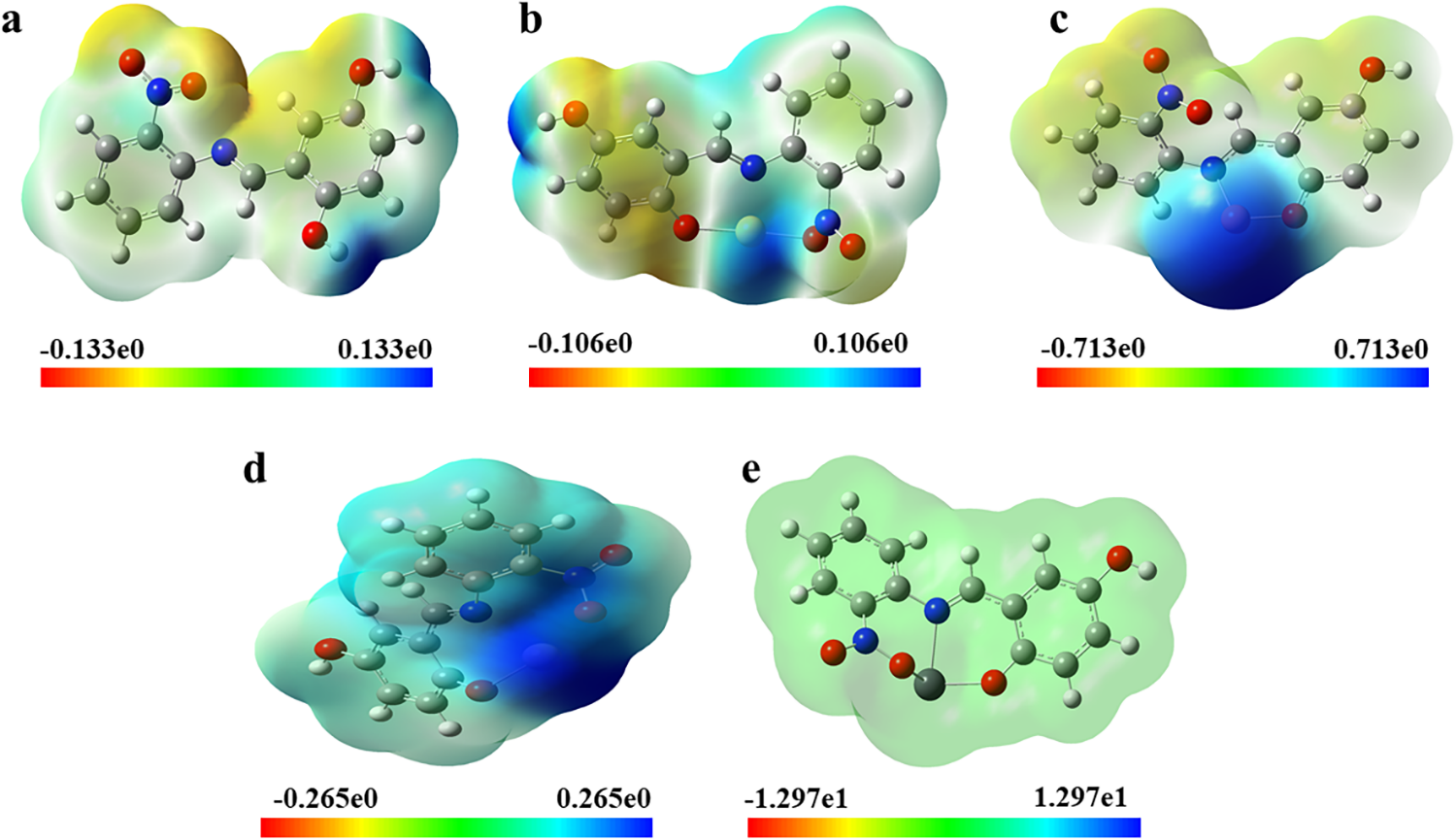
Molecular electrostatic potential maps of the (a) TSL1, (b) TSL1-Cd, (c) TSL1-Cu, (d) TSL1-Hg, and (e) TSL1-Pb.

The frontier molecular orbital (FMO) analysis of the free ligand TSL1 and its Cd­(II), Cu­(II), Hg­(II), and Pb­(II) complexes (Figure 19) offers a detailed understanding of how metal coordination reshapes the electronic structure and complements the trends inferred from the MEP surfaces. In all systems, the HOMO primarily represents the electron-donating regions of the ligand framework, whereas the LUMO highlights the most favorable electron-accepting domains; however, the spatial localization of these orbitals and their energetic separation are strongly dependent on the coordinated metal ion. For the free ligand TSL1 (Figure 19a), the HOMO (−8.1474 eV) is mainly distributed over the donor-rich segment of the molecule, involving the azomethine/thiosemicarbazone scaffold and adjacent π-systems, indicating the intrinsic electron-donating character of the ligand. The LUMO (−5.5235 eV) remains largely delocalized over the conjugated backbone, defining the preferred acceptor region. The resulting energy gap (Δ*E* = 2.6239 eV) is the largest within the series, suggesting comparatively higher electronic stability and a lower tendency toward charge redistribution relative to the coordinated systems. Upon Cd­(II) coordination (TSL1–Cd, Figure 19b), both frontier orbitals are modified through metal binding, and the electronic density becomes more evenly distributed across the coordination environment. The HOMO (−8.2032 eV) retains a predominantly ligand-centered nature, while the LUMO (−5.9627 eV) exhibits enhanced stabilization, consistent with an increased electron-accepting character upon complex formation. The reduced gap (Δ*E* = 2.2405 eV) relative to the free ligand indicates increased polarizability and an improved propensity for electronic transitions. In the Cu­(II) complex (TSL1–Cu, Figure 19c), the electronic structure is perturbed most strongly in energetic terms. Both the HOMO (−3.8133 eV) and LUMO (−3.1924 eV) are markedly shifted to higher energies compared to the other species, and the HOMO–LUMO separation becomes exceptionally small (Δ*E* = 0.6209 eV). The frontier orbital surfaces indicate intensified electronic coupling within the metal–ligand framework, implying a substantially enhanced charge-transfer character and the highest electronic softness among the studied systems. This exceptionally narrow gap is consistent with a highly responsive electronic system, which may facilitate electron redistribution during interactions such as coordination, adsorption, or redox-related processes. For the Hg­(II) complex (TSL1–Hg, Figure 19d), the HOMO (−8.2157 eV) remains largely associated with the ligand donor sites, while the LUMO (−6.0008 eV) is further stabilized, reflecting efficient ligand-to-metal electronic communication typical of soft metal–soft donor interactions. The corresponding gap (Δ*E* = 2.2150 eV) is smaller than that of the free ligand but comparable to those of the Cd­(II) and Pb­(II) complexes, indicating intermediate electronic flexibility. A similar intermediate behavior is observed for the Pb­(II) complex (TSL1–Pb, Figure 19e). The HOMO (−8.4886 eV) is slightly more stabilized than in the Cd­(II) and Hg­(II) analogues, and the LUMO (−6.3102 eV) is also stabilized, yielding Δ*E* = 2.1785 eV. This value indicates that Pb­(II) coordination enhances electronic softness relative to the free ligand, yet it does not exceed the Cu­(II) complex in charge-transfer propensity. The orbital distributions further suggest that metal binding contributes to a broader redistribution of electron density within the coordination framework, consistent with the distinct electronic environment associated with Pb­(II). Overall, the FMO results establish a clear structure–reactivity relationship governed primarily by the HOMO–LUMO gaps. The free ligand exhibits the largest Δ*E* and thus the highest electronic stability, whereas complex formation generally reduces Δ*E*, indicating enhanced polarizability. Among the complexes, the Cu­(II) derivative shows an exceptionally small gap and therefore the strongest charge-transfer character and highest predicted reactivity. Based on the calculated Δ*E* values, the electronic softness (reactivity) trend can be expressed as TSL1–Cu ≫ TSL1–Pb ≈ TSL1–Hg ≈ TSL1–Cd > TSL1, in strong agreement with the metal-induced polarization patterns observed in the MEP analysis.^48^

**Figure 19:**
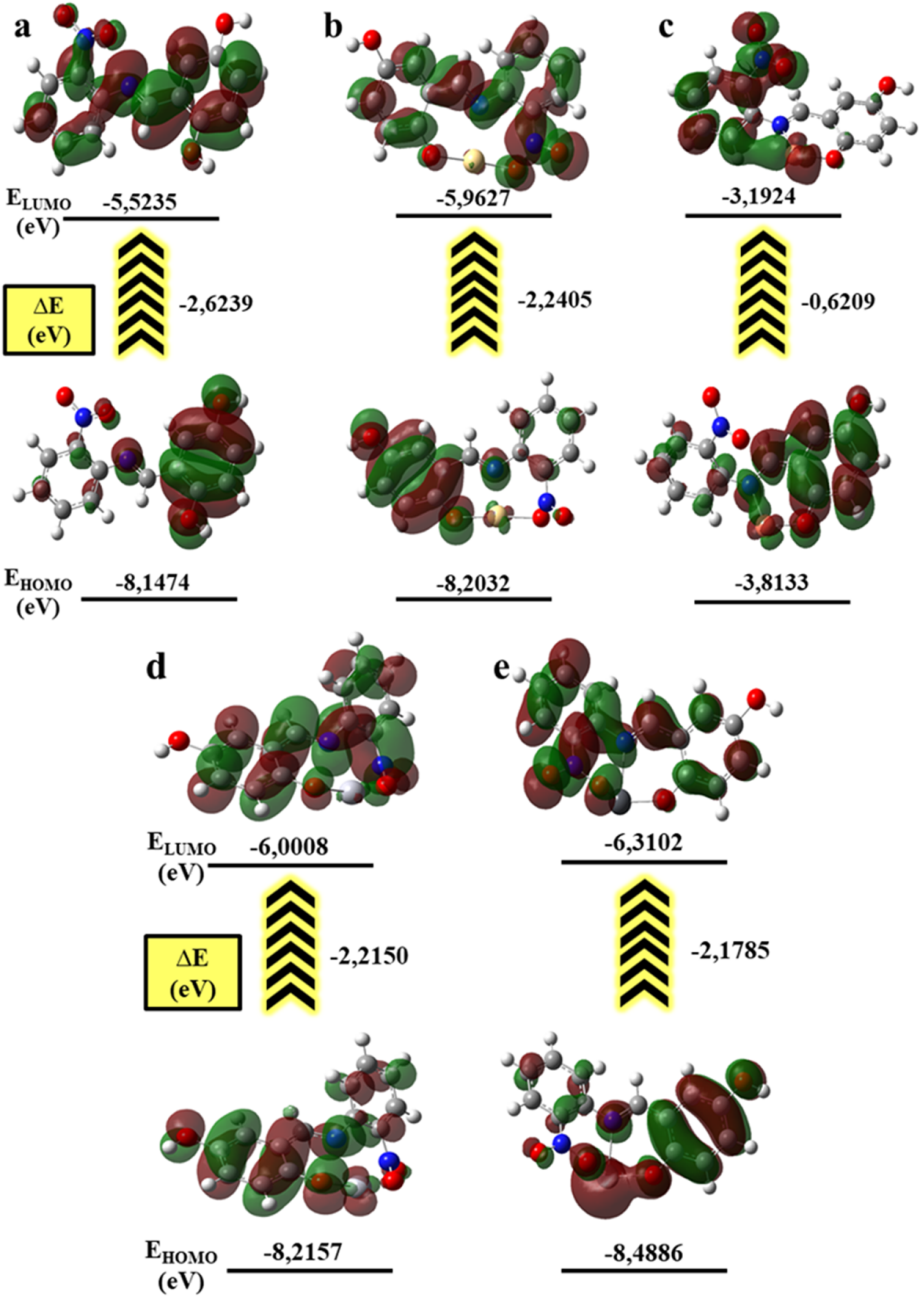
Frontier molecule orbitals and energy gaps of (a) TSL1, (b) TSL1-Cd, (c) TSL1-Cu, (d) TSL1-Hg, and (e) TSL1-Pb.

Quantitatively, the calculated frontier orbital energies (eV) and the associated HOMO–LUMO gaps (Δ*E* = |*E*_HOMO_ – *E*_LUMO_|) for TSL1 and its metal complexes follow a clear trend (Table 13). The free ligand TSL1 exhibits *E*_HOMO_/*E*_LUMO_ values of −8.1474/–5.5235 eV, corresponding to the largest gap (Δ*E* ≈ 2.6239 eV). Upon coordination, the energy gap decreases for all complexes, with TSL1–Cu displaying a remarkably narrow gap (Δ*E* ≈ 0.6209 eV), indicating substantially enhanced polarizability and charge-redistribution capability relative to the other species. The remaining complexes show intermediate gaps, clustered in a narrow range (TSL1–Cd: 2.2405 eV; TSL1–Hg: 2.2150 eV; TSL1–Pb: 2.1785 eV). Accordingly, the Δ*E*-derived electronic reactivity (softness) order can be expressed as TSL1–Cu ≫ TSL1–Pb ≳ TSL1–Hg ≳ TSL1–Cd > TSL1, which is consistent with the metal-induced polarization trends inferred from the MEP surfaces. Global reactivity descriptors derived from Koopmans-type relationships further support these observations (Table 1). The ionization potentials (IP), taken as |*E*_HOMO_|, increase in the order TSL1–Cu (3.8133 eV) ≪ TSL1 (8.1474 eV) < TSL1–Cd (8.2032 eV) ≈ TSL1–Hg (8.2157 eV) < TSL1–Pb (8.4886 eV), suggesting that electron removal is energetically most facile for the Cu­(II) complex—consistent with its exceptionally small Δ*E* and high electronic responsiveness. Electron affinities (EA ≈ |*E*_LUMO_|) follow a similar stabilization pattern, with the lowest EA observed again for TSL1–Cu (3.1924 eV), while the remaining complexes exhibit higher EA values (≈5.52–6.31 eV), indicative of stronger electron-accepting stabilization upon complexation. Electronegativity values (χ = (IP + EA)/2) are markedly lower for TSL1–Cu (3.5028 eV) compared with the other members (≈6.84–7.40 eV), reflecting a distinct electronic regime for the Cu­(II) complex in which both frontier levels are shifted and the system becomes exceptionally “soft” (η = Δ*E*/2 = 0.3105 eV). In contrast, the remaining complexes retain hardness values close to ∼1.09–1.12 eV, while the free ligand shows the highest hardness (1.3120 eV), in agreement with its larger HOMO–LUMO separation and greater kinetic stability. Importantly, the global electrophilicity index (ω = χ^2^/2η) highlights a complementary trend: TSL1–Pb (25.1327) > TSL1–Hg (22.8119) ≳ TSL1–Cd (22.3912) > TSL1–Cu (19.7598) > TSL1 (17.8067) (Table 13). This indicates that, despite the extreme softness of TSL1–Cu (small η), its comparatively low χ limits ω, whereas Pb­(II) coordination yields the most electrophilic system due to the combined effect of high χ and moderate η. Overall, the joint consideration of Δ*E*/η (softness-reactivity) and ω (electrophilic driving force) confirms that metal coordination systematically governs the electronic reactivity profile of the TSL1 framework, in line with the trends suggested by the MEP and FMO analyses.

**Table 13:** Chemical Parameters of the TSL1, TSL1-Cd, TSL1-Cu, TSL1-Hg, and TSL1-Pb

chemical parameters	TSL1	TSL1-Cd	TSL1-Cu	TSL1-Hg	TSL1-Pb
*E*_HOMO_	–8,1474	–8.2032	–3.8133	–8.2157	–8.4886
*E*_LUMO_	–5.5235	–5.9627	–3.1924	–6.0008	–6.3102
Δ*E*_energy gap_	–2.6239	–2.2405	–0.6209	–2.2150	–2.1785
Ionization Potential (IP)	8.1474	8,2032	3.8133	8.2157	8.4886
Electron Affinity (EA)	5.5235	5,9627	3.1924	6.0008	6.3102
Electronegativity (χ)	6.8355	7.0829	3.5028	7.1083	7.3994
Hardness (η)	1.3120	1.1203	0.3105	1.1075	1.0892
Softness (σ)	282.1809	330.4676	1192.4058	334.2838	339.8788
Chemical Potential (μ)	–6.8355	–7.0829	–3.5028	–7.1083	–7.3994
Global Electrophilicity (ω)	17.8067	22.3912	19.7598	22.8119	25.1327

### Toxicity

In silico toxicity assessment of TSL1 was performed using the ProTox platform, and the predicted endpoints are summarized in Table 14 and visualized in the toxicity radar (Figure 20). The model predicts an LD_50_ value of 1345 mg·kg^–1^, representing low acute toxicity and indicating a suitable preliminary safety margin for laboratory procedures and analytical applications performed at trace exposure levels. Organ-specific toxicity assessment reveals a selective profile rather than generalized systemic responsibility. TSL1 is predicted to exhibit hepatotoxicity (Active, probability 0.53), while neurotoxicity, nephrotoxicity, respiratory toxicity, and cardiotoxicity are all predicted to be inactive at moderate to high confidence levels (probability values of 0.76, 0.53, 0.67, and 0.64, respectively). This distribution suggests that potential adverse effects are more likely to be localized and organ-specific rather than widespread multiorgan toxicity, a feature advantageous for materials intended for controlled analytical use. In terms of additional toxicological endpoints, TSL1 is estimated to be inactive with respect to immunotoxicity (0.59) and cytotoxicity (0.74), suggesting a low probability of nonspecific immunity or cellular damage. In terms of exposure, the compound is estimated to be impermeable to the blood-brain barrier (Inactive, 0.51), indicating a reduced risk of central nervous system involvement and representing an additional positive safety feature. In total, the toxicity radar (Figure 20) shows a profile characterized by low acute toxicity, absence of widespread organ toxicity, absence of cytotoxic and immunotoxic effects, and impermeability to the blood-brain barrier. In the context of its intended use as a sorbent material in marine and environmental metal analysis, where its use is limited to solid-phase, recoverable, and reusable formats, these in silico findings support the practical suitability and controlled safety of TSL1.

**Figure 20:**
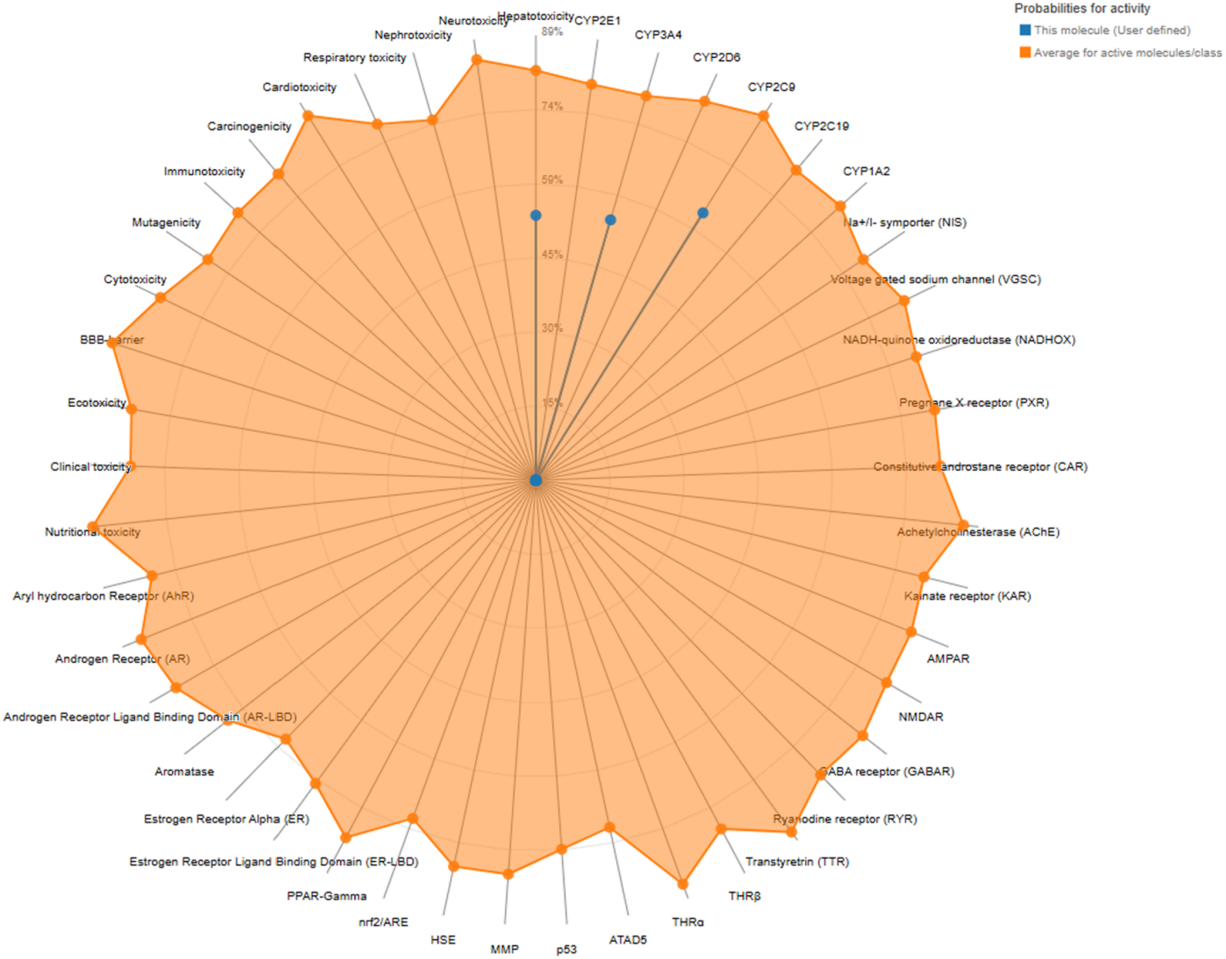
Toxicity radar of TSL1.

**Table 14:** Toxicity Level of TSL1 (Probability of Toxicity Is Shown in Brackets)

Derivatives	Pred. LD50 (mg/kg)	Hepatotoxicity	Neurotoxicity	Nephrotoxicity	Respiratory toxicity	Cardiotoxicity	Immunotoxicity	Cytotoxicity	BBB-barrier
TSL1	1345	Active (0.53)	Inactive (0.76)	Inactive (0.53)	Inactive (0.67)	Inactive (0.64)	Inactive (0.59)	Inactive (0.74)	Inactive (0.51)

## Conclusion

In this work, a novel dihydroxyphenyl–nitroaryl azomethine Schiff base ligand (TSL1) was successfully synthesized and employed as a functional ligand for the preparation of a silica-based sorbent designed for ultrasound-assisted dispersive micro-solid-phase extraction. Comprehensive spectroscopic, morphological, and textural characterization confirmed the successful immobilization of TSL1 onto the silica surface while preserving its coordination functionality. The optimized UA-DMSPE procedure enabled efficient and selective extraction of Hg­(II), Cu­(II), Pb­(II), and Cd­(II) ions, providing high recoveries, low detection limits, and excellent precision. Statistical evaluation using one-way ANOVA verified the robustness of the optimized parameters, with solution pH and sorbent amount identified as key factors influencing extraction performance.

Application to real seawater and mussel samples demonstrated reliable performance with controlled matrix effects under saline and biological conditions. Complementary computational analyses were conducted to provide molecular-level support for the observed extraction behavior, while preliminary in silico toxicity evaluation and green analytical metrics further confirmed the suitability and environmental compatibility of the developed method.

Overall, the TSL1-functionalized silica sorbent combined with UA-DMSPE represents a sensitive, selective, and environmentally sustainable analytical strategy for trace metal determination in complex marine and food-related matrices, offering a promising foundation for further development of Schiff base–based sorbent systems in environmental monitoring and food safety applications.
